# Supplementary material for: Design, Synthesis and Antitumor Activity of Quercetin Derivatives Containing a Quinoline Moiety
Source: Molecules. 2024 Jan 2;29(1):240. doi: 10.3390/molecules29010240 (PMC10780609; doi:10.3390/molecules29010240)
Supplement: Supplementary file 1 [file molecules-29-00240-s001.zip › molecules-2696444-supplementary.pdf]

Supporting Information

Table of contents

1	Compounds characterization.	Pages 2-31

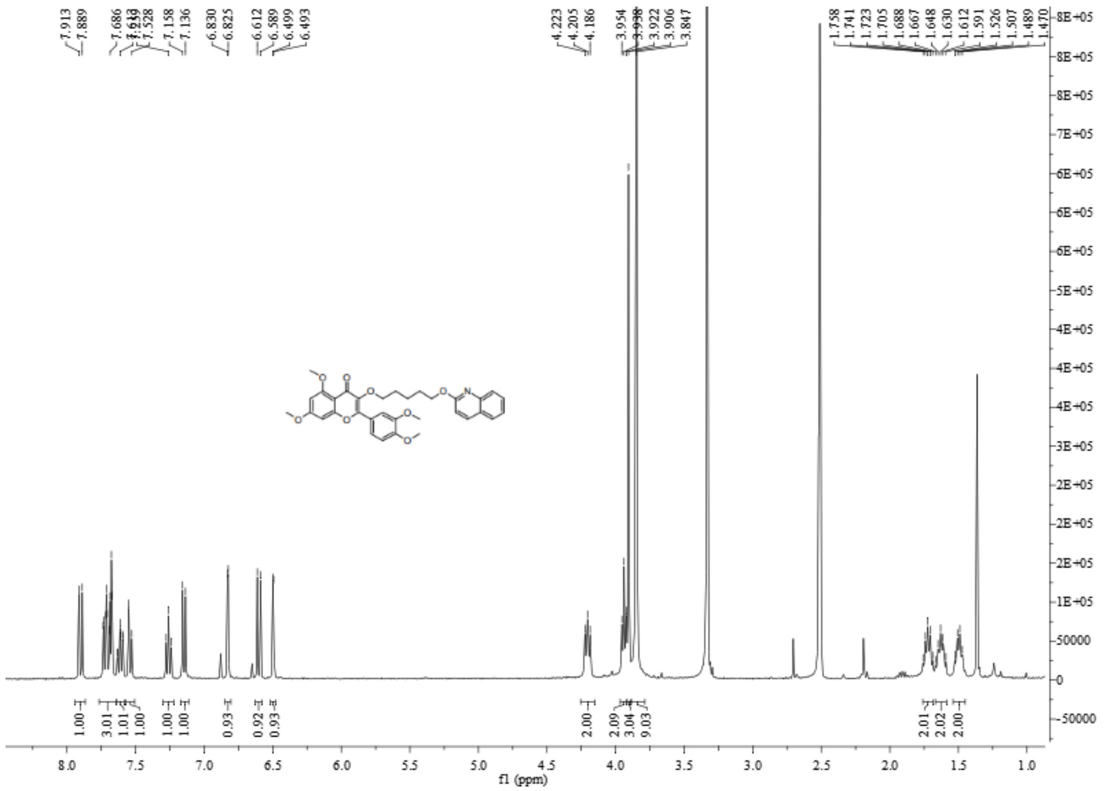

^{1}H-NMR spectrum of 2-(3,4-dimethoxyphenyl)-5,7-dimethoxy-3-((5-(quinolin-2-yloxy)pentyl)oxy)-4*H*-chromen-4-one (**3a**)

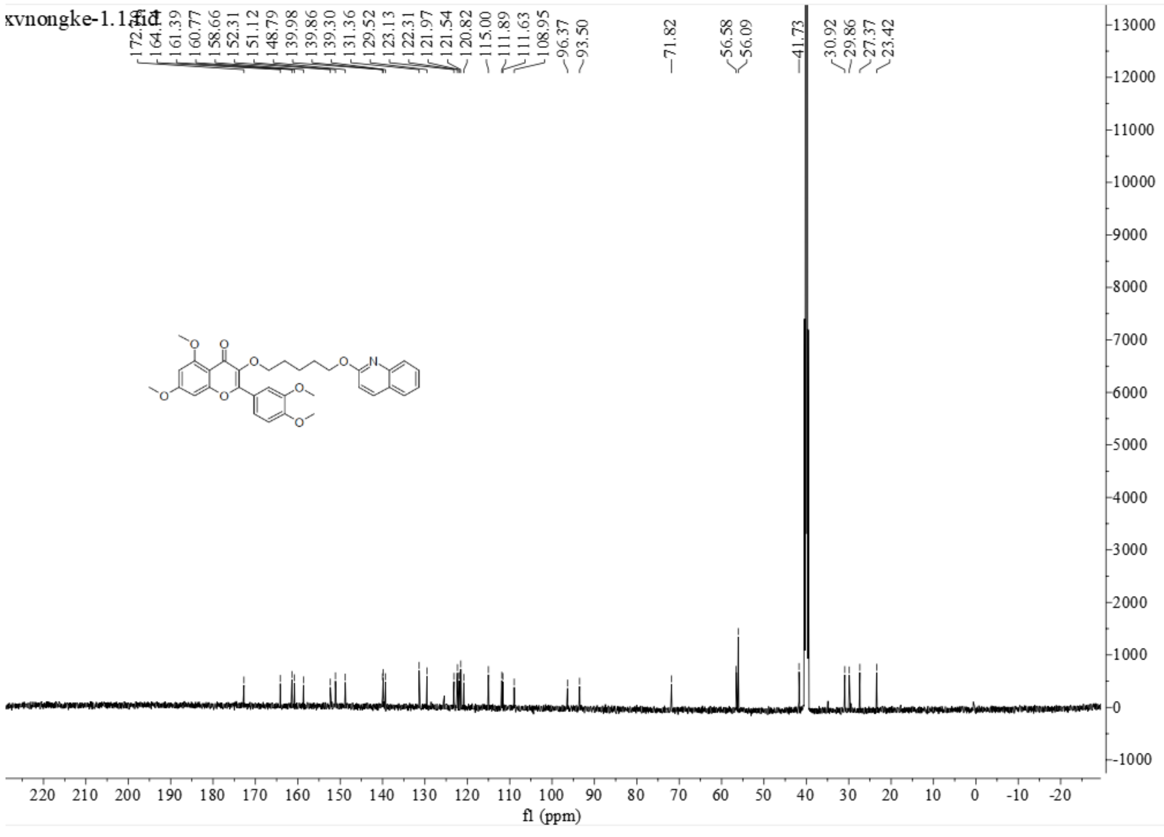

^{13}C-NMR spectrum of 2-(3,4-dimethoxyphenyl)-5,7-dimethoxy-3-((5-(quinolin-2-yloxy)pentyl)oxy)-4*H*-chromen-4-one (**3a**)

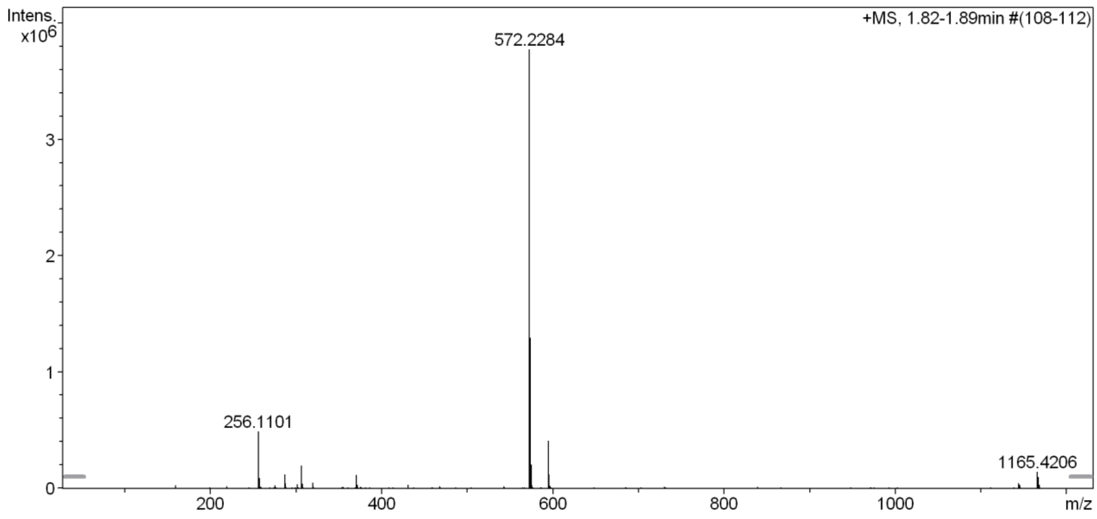

HRMS spectrum of 2-(3,4-dimethoxyphenyl)-5,7-dimethoxy-3-((5-(quinolin-2-yloxy)pentyl)oxy)-4*H*-chromen-4-one (**3a**)

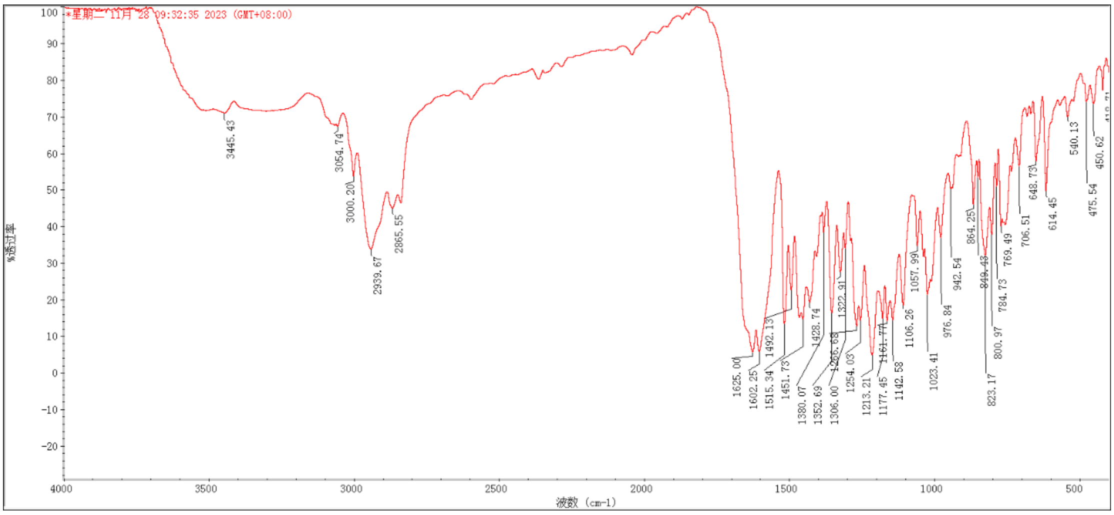

IR spectra of 2-(3,4-dimethoxyphenyl)-5,7-dimethoxy-3-((5-(quinolin-2-yloxy)pentyl)oxy)-4*H*-chromen-4-one (**3a**)

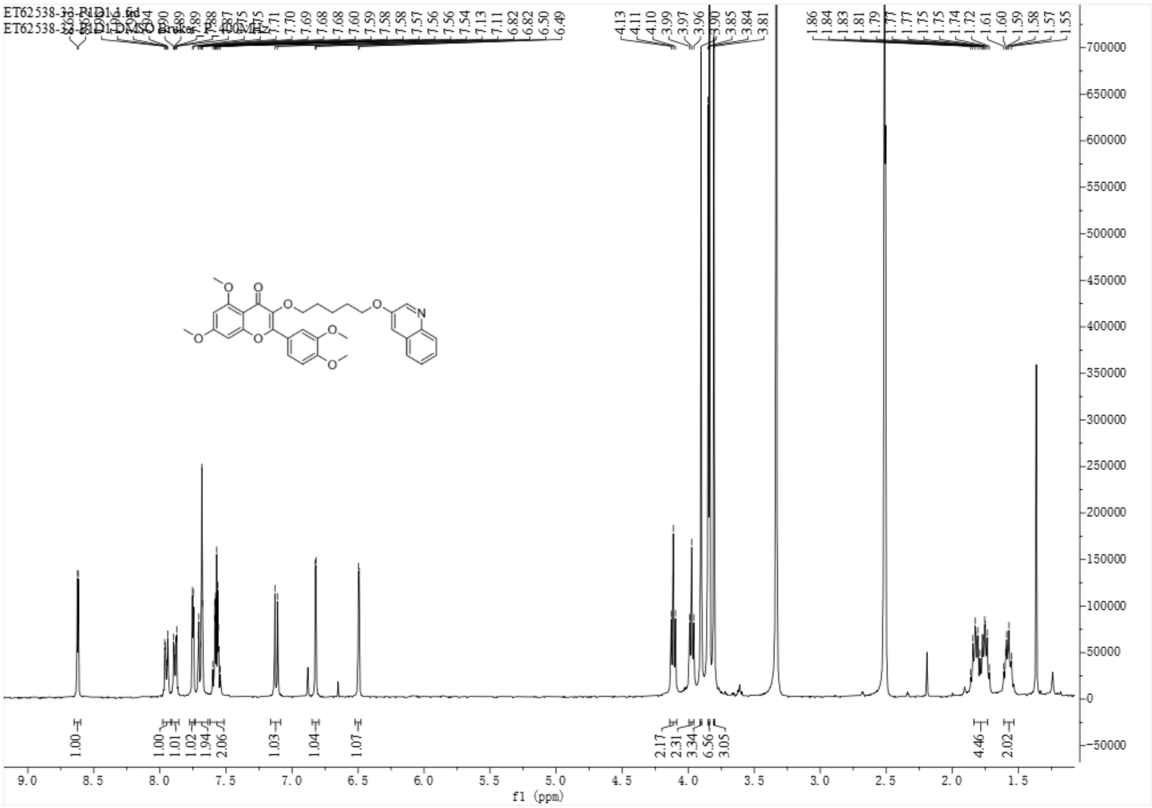

^{1}H-NMR spectrum of 2-(3,4-dimethoxyphenyl)-5,7-dimethoxy-3-((5-(quinolin-3-yloxy)pentyl)oxy)-4*H*-chromen-4-one (**3b**)

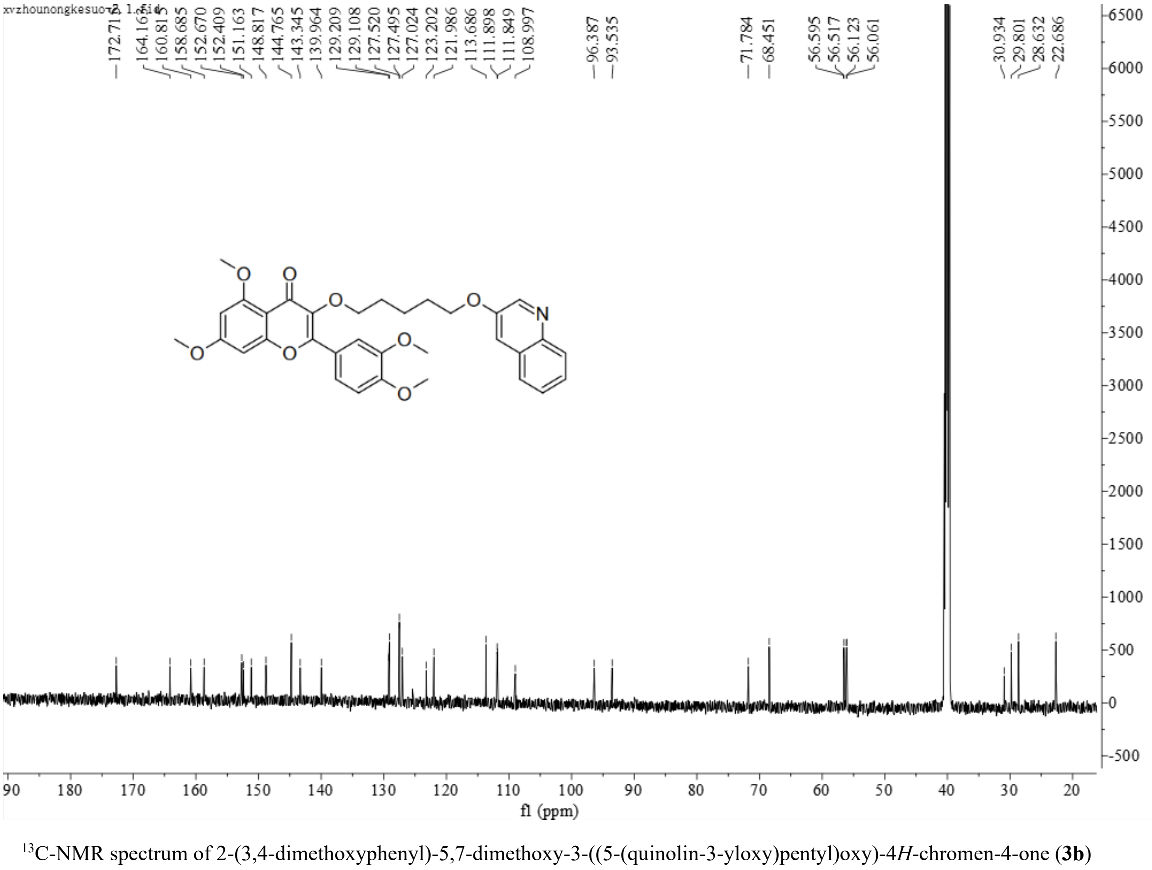

^{13}C-NMR spectrum of 2-(3,4-dimethoxyphenyl)-5,7-dimethoxy-3-((5-(quinolin-3-yloxy)pentyl)oxy)-4*H*-chromen-4-one (**3b**)

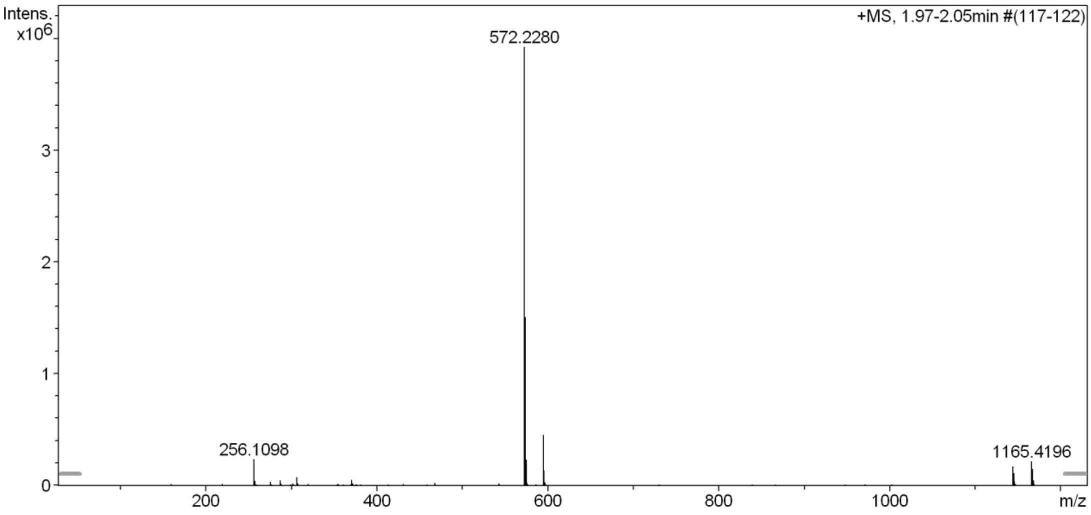

HRMS spectrum of 2-(3,4-dimethoxyphenyl)-5,7-dimethoxy-3-((5-(quinolin-3-yloxy)pentyl)oxy)-4*H*-chromen-4-one (**3b**)

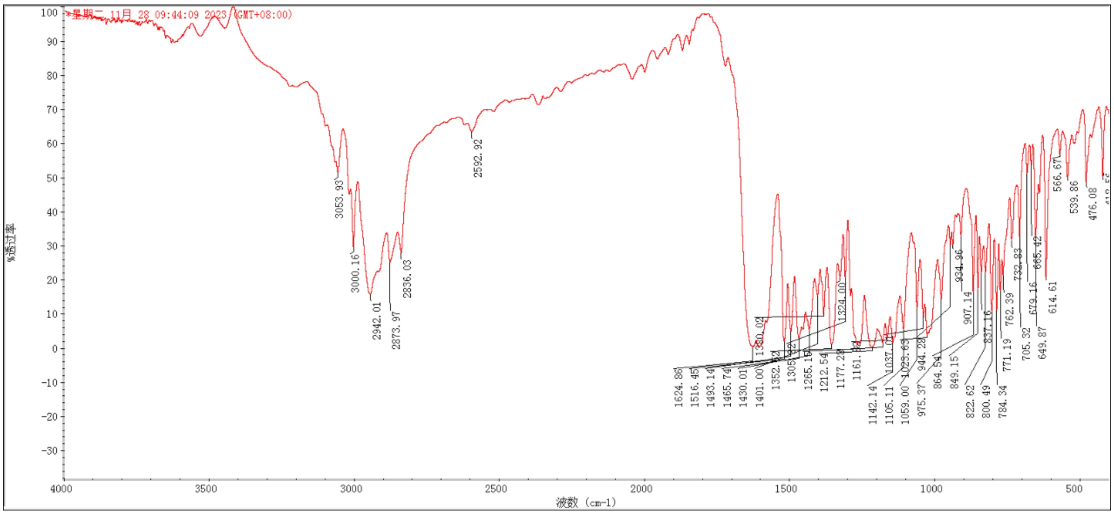

IR spectra of 2-(3,4-dimethoxyphenyl)-5,7-dimethoxy-3-((5-(quinolin-3-yloxy)pentyl)oxy)-4*H*-chromen-4-one (**3b**)

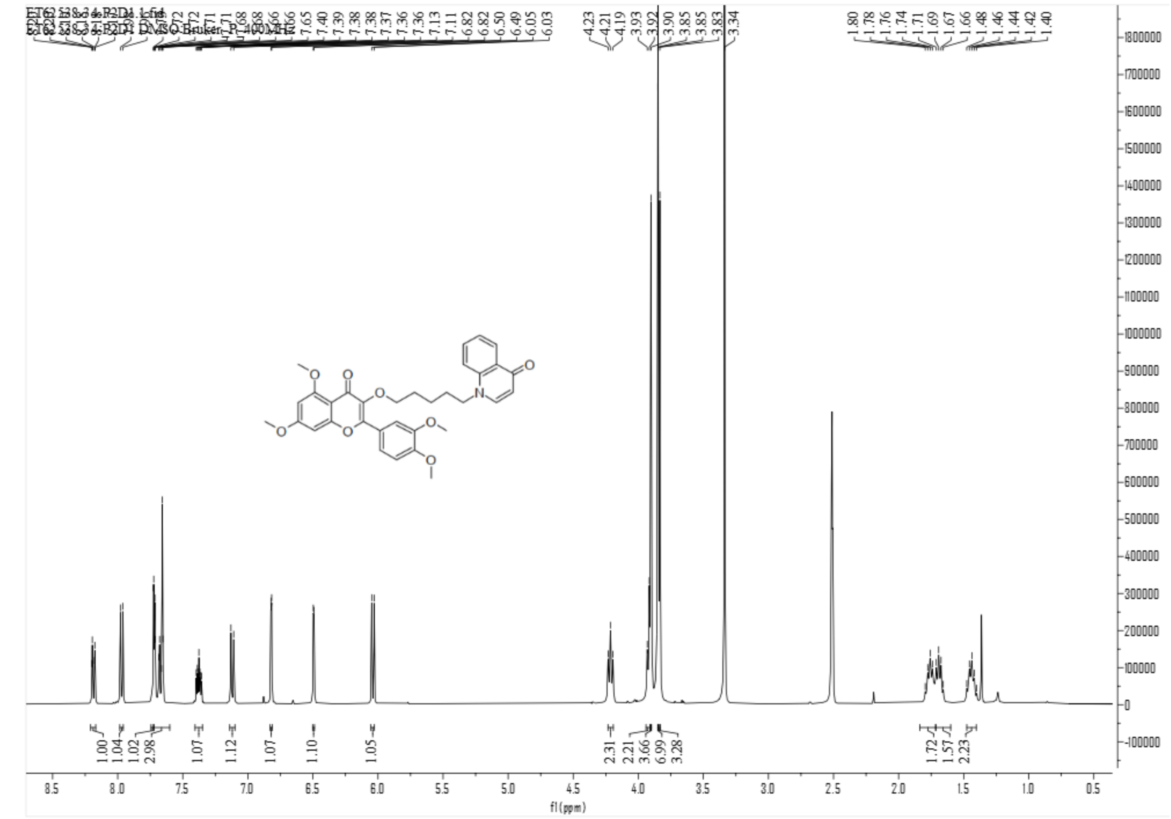

^{1}H-NMR spectrum of 2-(3,4-dimethoxyphenyl)-5,7-dimethoxy-3-((5-(quinolin-4-yloxy)pentyl)oxy)-4H-chromen-4-one (**3c**)

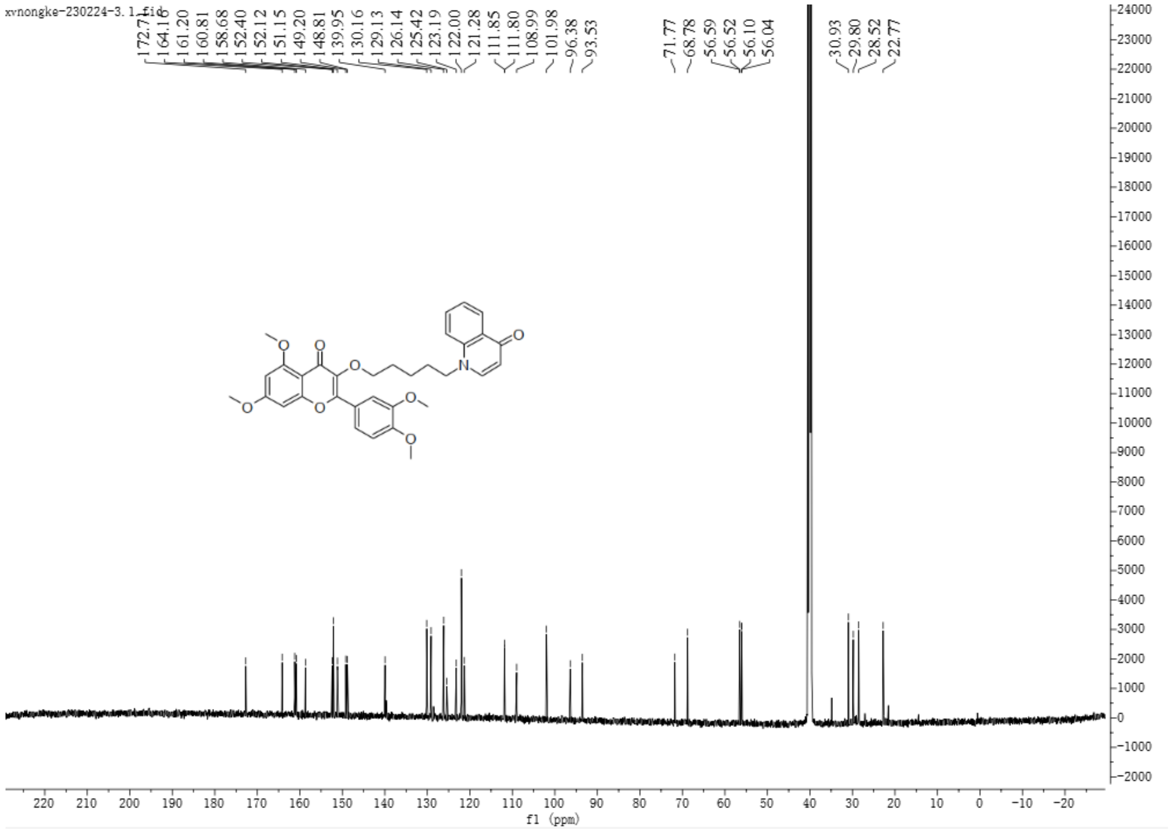

^{13}C-NMR spectrum of 2-(3,4-dimethoxyphenyl)-5,7-dimethoxy-3-((5-(quinolin-4-yloxy)pentyl)oxy)-4H-chromen-4-one (**3c**)

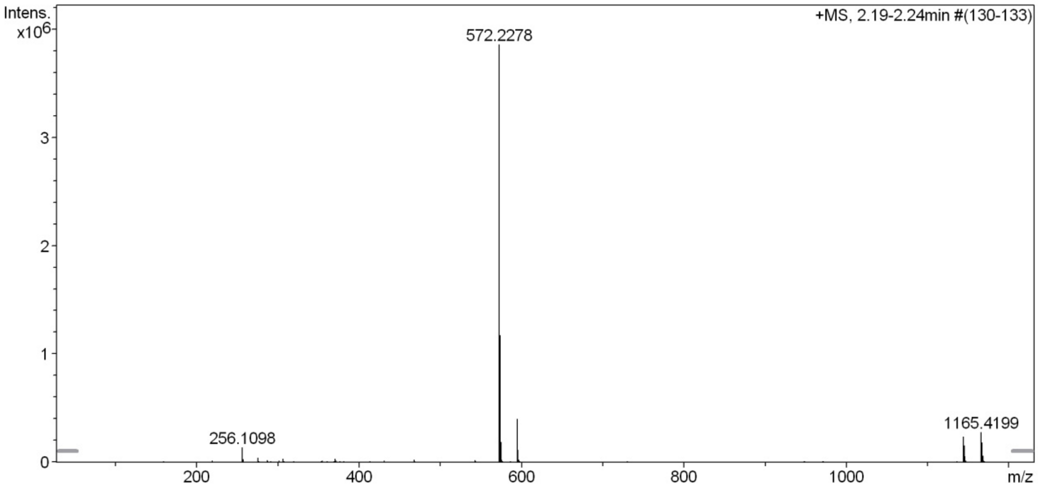

HRMS spectrum of 2-(3,4-dimethoxyphenyl)-5,7-dimethoxy-3-((5-(quinolin-4-yloxy)pentyl)oxy)-4H-chromen-4-one (**3c**)

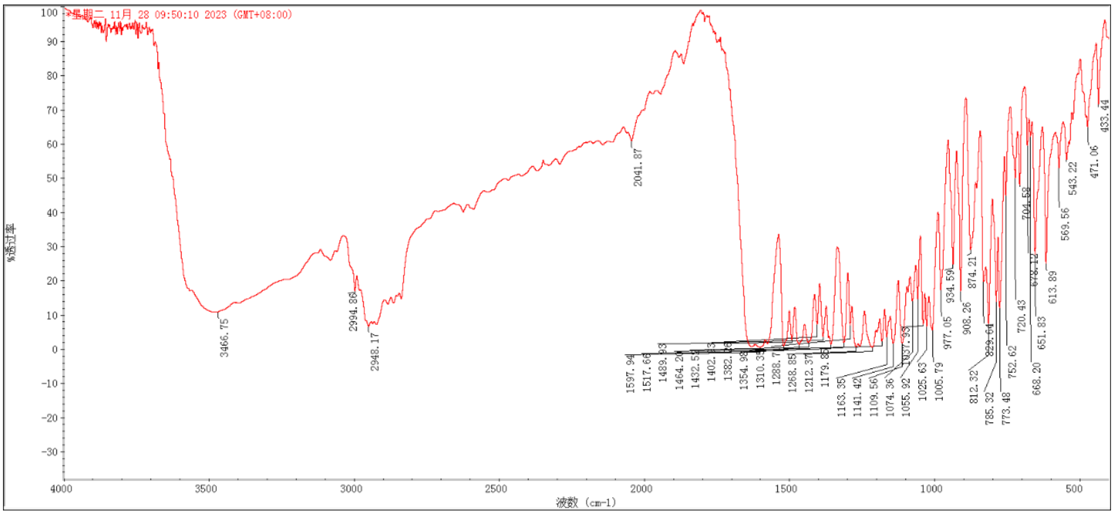

IR spectra of 2-(3,4-dimethoxyphenyl)-5,7-dimethoxy-3-((5-(quinolin-4-yloxy)pentyl)oxy)-4H-chromen-4-one (**3c**)

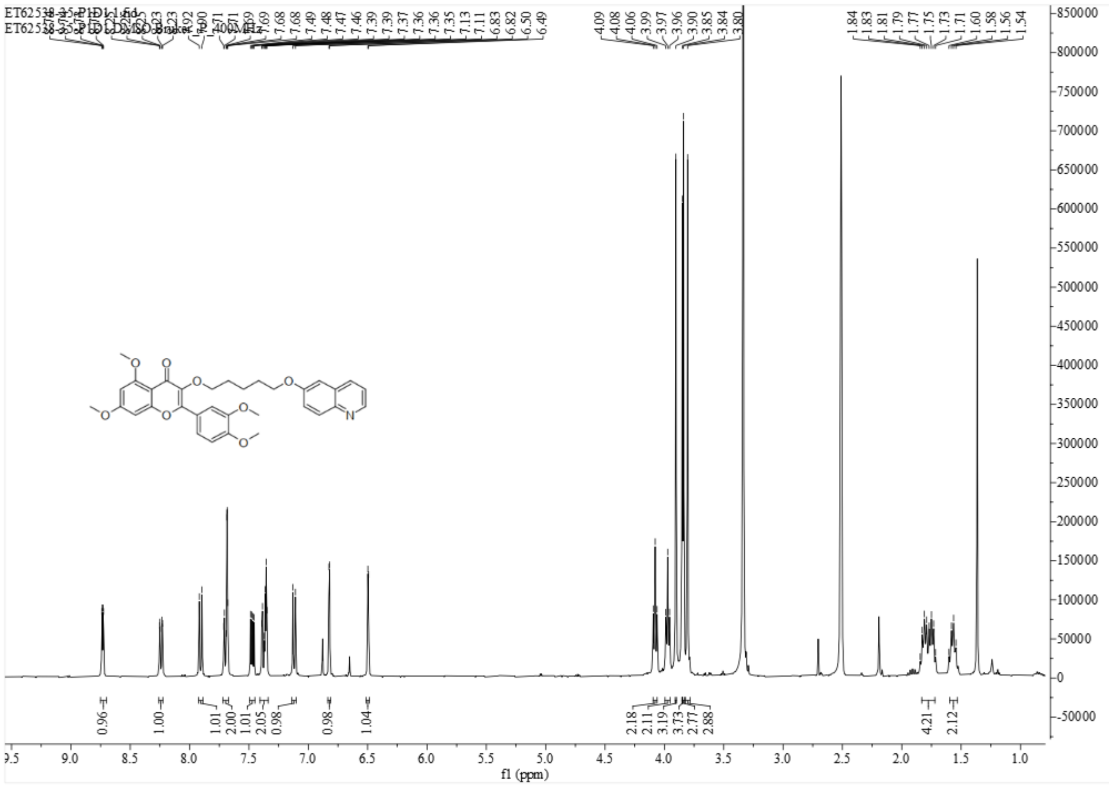

^{1}H-NMR spectrum of 2-(3,4-dimethoxyphenyl)-5,7-dimethoxy-3-((5-(quinolin-6-yloxy)pentyl)oxy)-4H-chromen-4-one **(3d)**

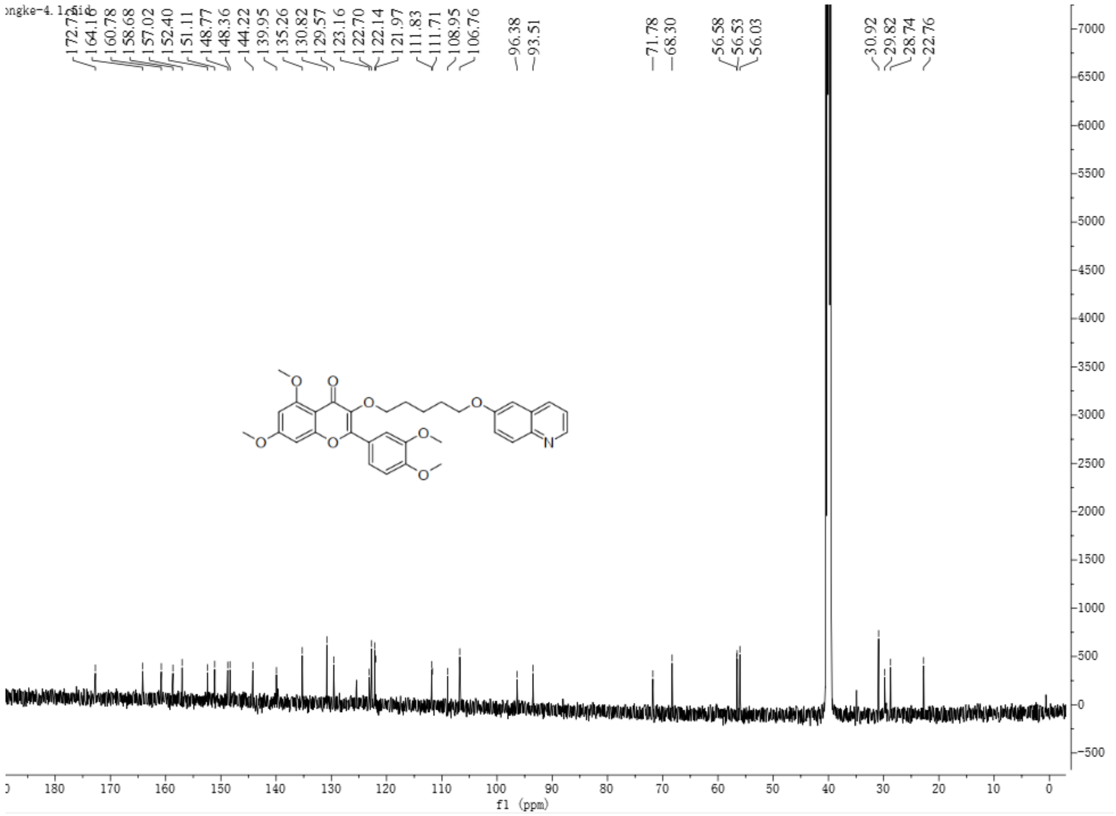

^{13}C-NMR spectrum of 2-(3,4-dimethoxyphenyl)-5,7-dimethoxy-3-((5-(quinolin-6-yloxy)pentyl)oxy)-4H-chromen-4-one **(3d)**

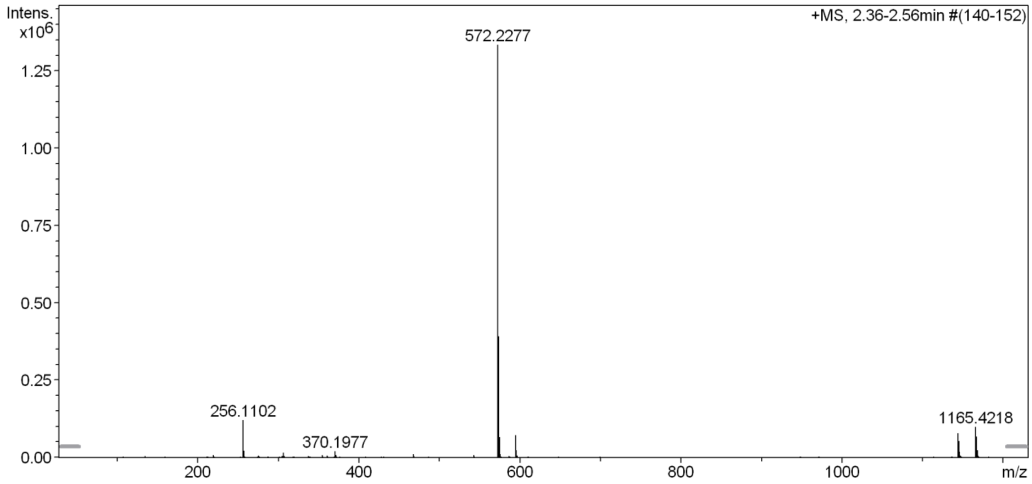

HRMS spectrum of 2-(3,4-dimethoxyphenyl)-5,7-dimethoxy-3-((5-(quinolin-6-yloxy)pentyl)oxy)-4H-chromen-4-one **(3d)**

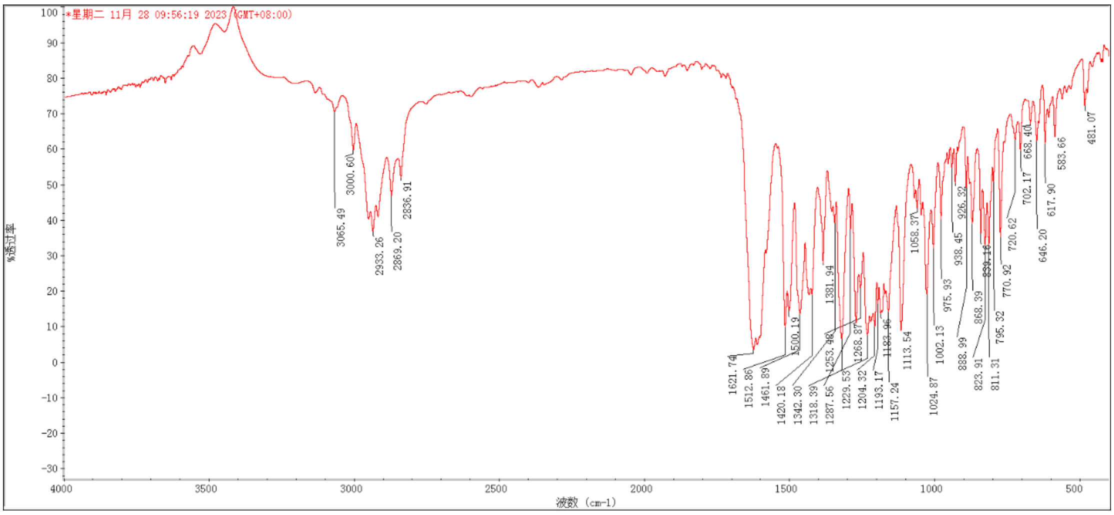

IR spectra of 2-(3,4-dimethoxyphenyl)-5,7-dimethoxy-3-((5-(quinolin-6-yloxy)pentyl)oxy)-4H-chromen-4-one **(3d)**

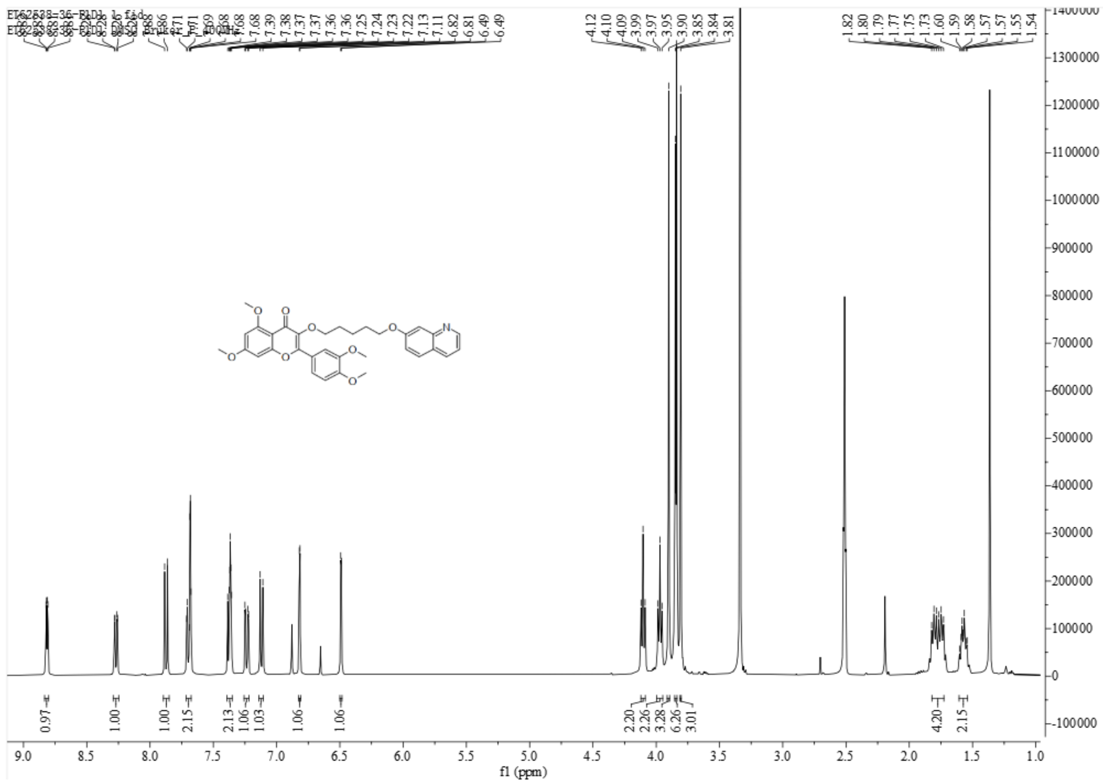

^{1}H-NMR spectrum of 2-(3,4-dimethoxyphenyl)-5,7-dimethoxy-3-((5-(quinolin-7-yloxy)pentyl)oxy)-4H-chromen-4-one (**3e**)

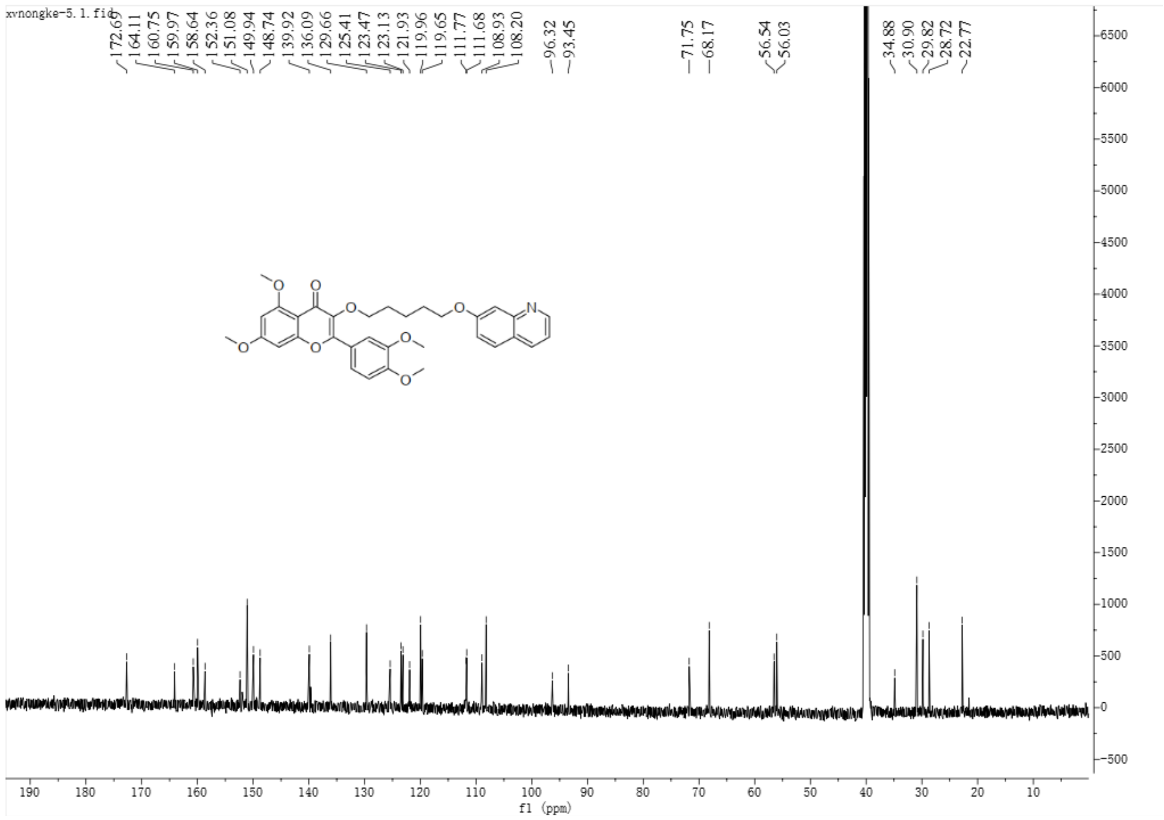

^{13}C-NMR spectrum of 2-(3,4-dimethoxyphenyl)-5,7-dimethoxy-3-((5-(quinolin-7-yloxy)pentyl)oxy)-4H-chromen-4-one (**3e**)

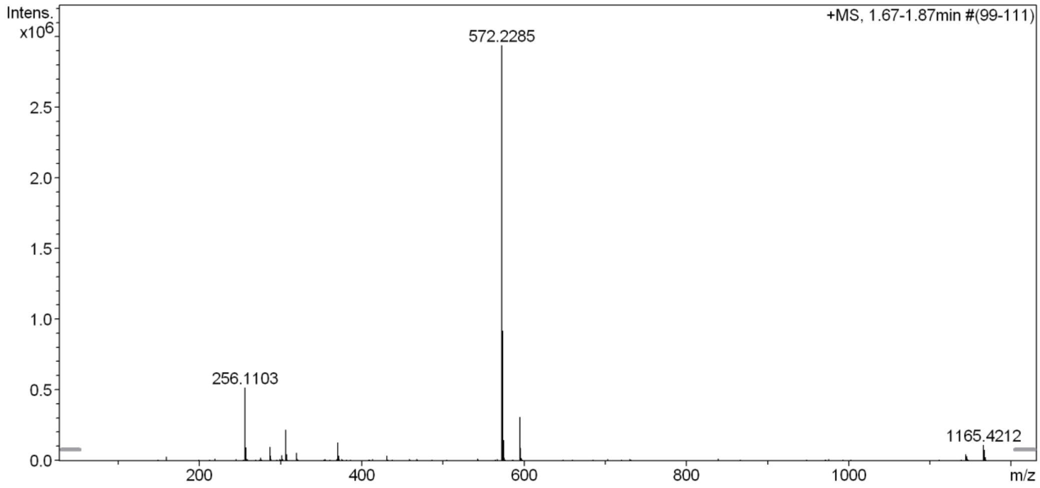

HRMS spectrum of 2-(3,4-dimethoxyphenyl)-5,7-dimethoxy-3-((5-(quinolin-7-yloxy)pentyl)oxy)-4H-chromen-4-one (**3e**)

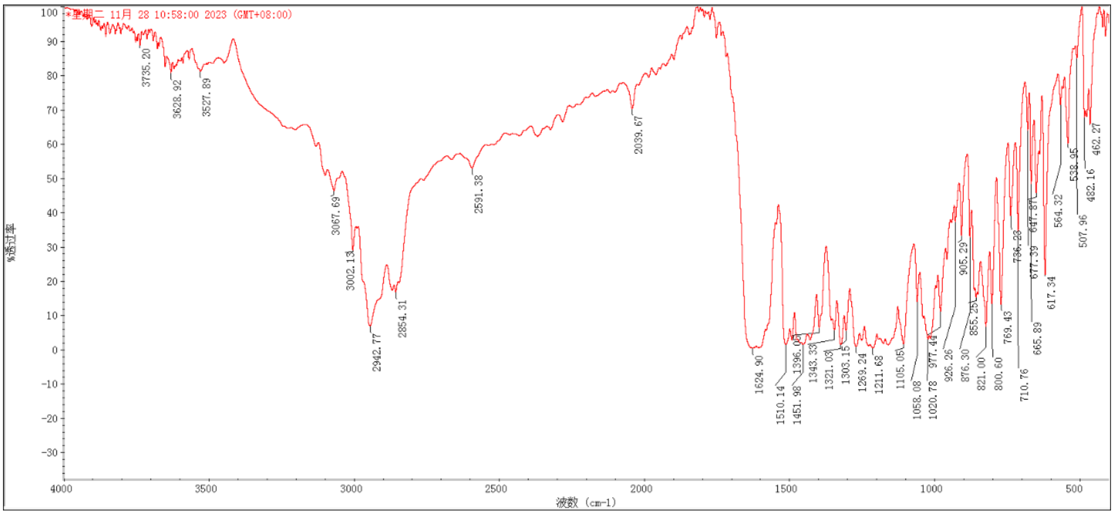

IR spectra of 2-(3,4-dimethoxyphenyl)-5,7-dimethoxy-3-((5-(quinolin-7-yloxy)pentyl)oxy)-4H-chromen-4-one (**3e**)

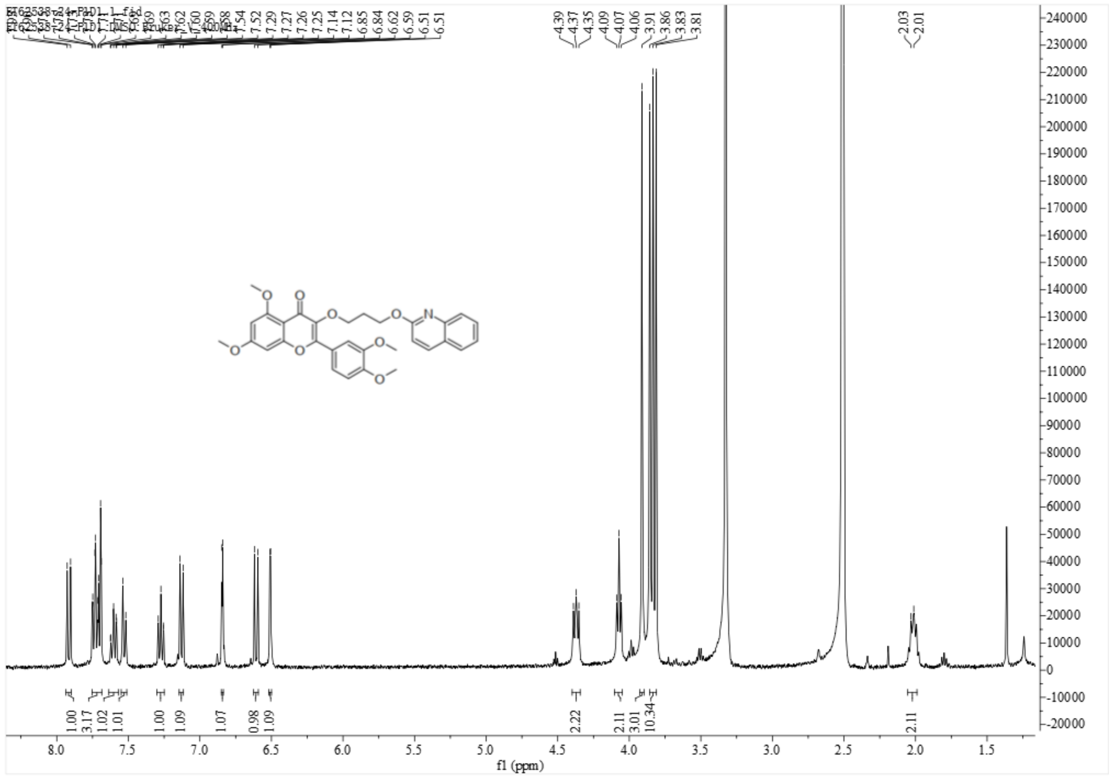

^{1}H-NMR spectrum of 2-(3,4-dimethoxyphenyl)-5,7-dimethoxy-3-(3-(quinolin-2-yloxy)propoxy)-4H-chromen-4-one (**3f**)

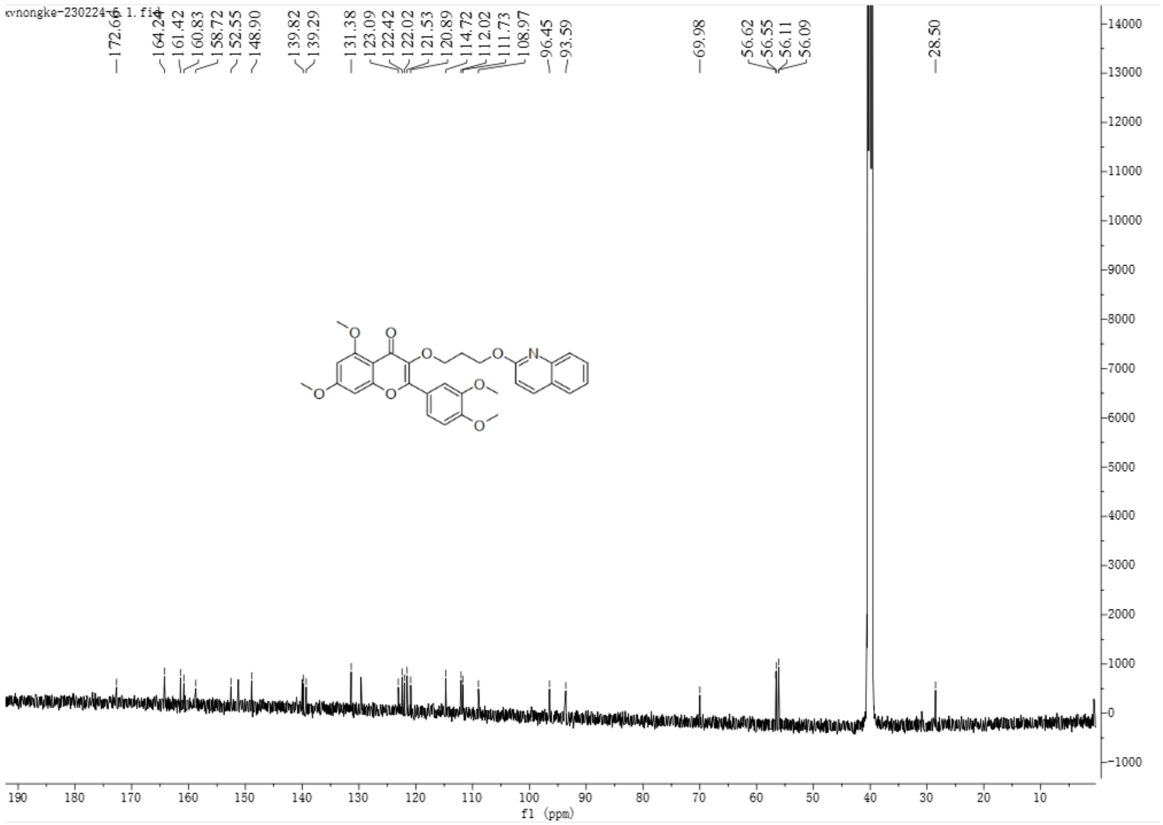

^{13}C-NMR spectrum of 2-(3,4-dimethoxyphenyl)-5,7-dimethoxy-3-(3-(quinolin-2-yloxy)propoxy)-4H-chromen-4-one (**3f**)

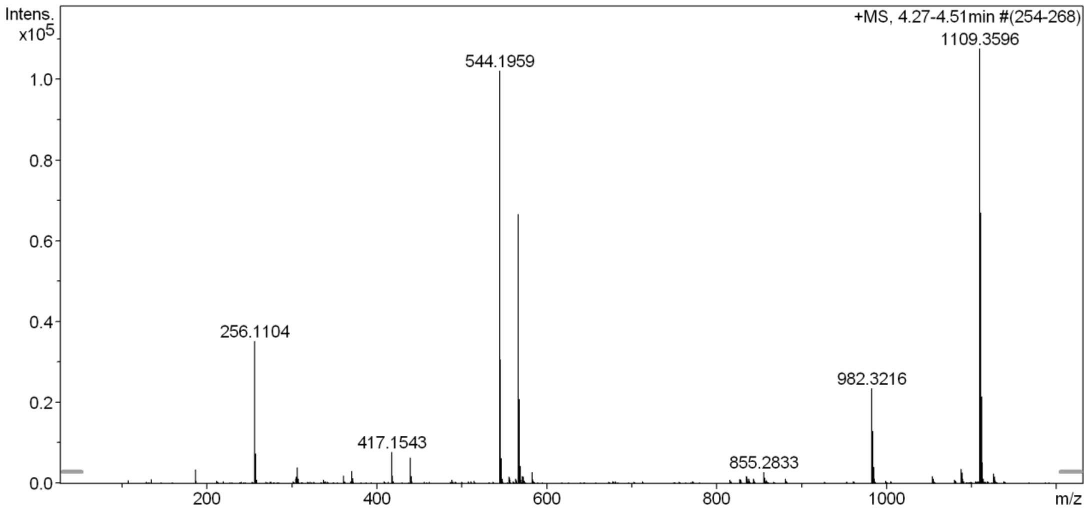

HRMS spectrum of 2-(3,4-dimethoxyphenyl)-5,7-dimethoxy-3-(3-(quinolin-2-yloxy)propoxy)-4H-chromen-4-one (**3f**)

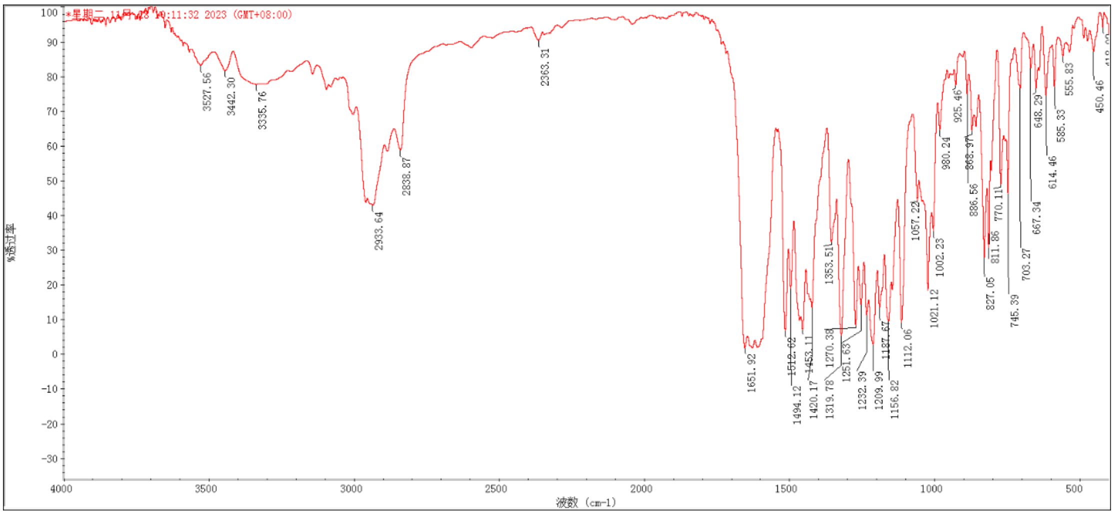

IR spectra of 2-(3,4-dimethoxyphenyl)-5,7-dimethoxy-3-(3-(quinolin-2-yloxy)propoxy)-4H-chromen-4-one (**3f**)

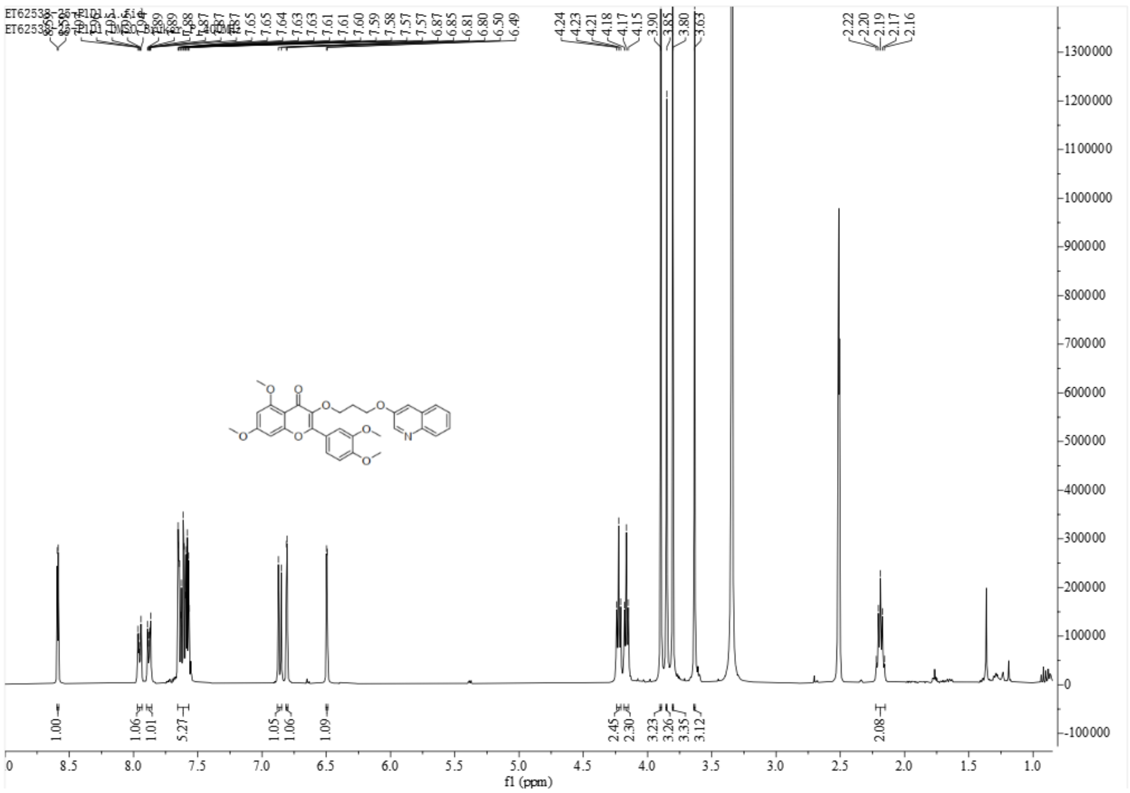

^{1}H-NMR spectrum of 2-(3,4-dimethoxyphenyl)-5,7-dimethoxy-3-(3-(quinolin-3-yloxy)propoxy)-4H-chromen-4-one (**3g**)

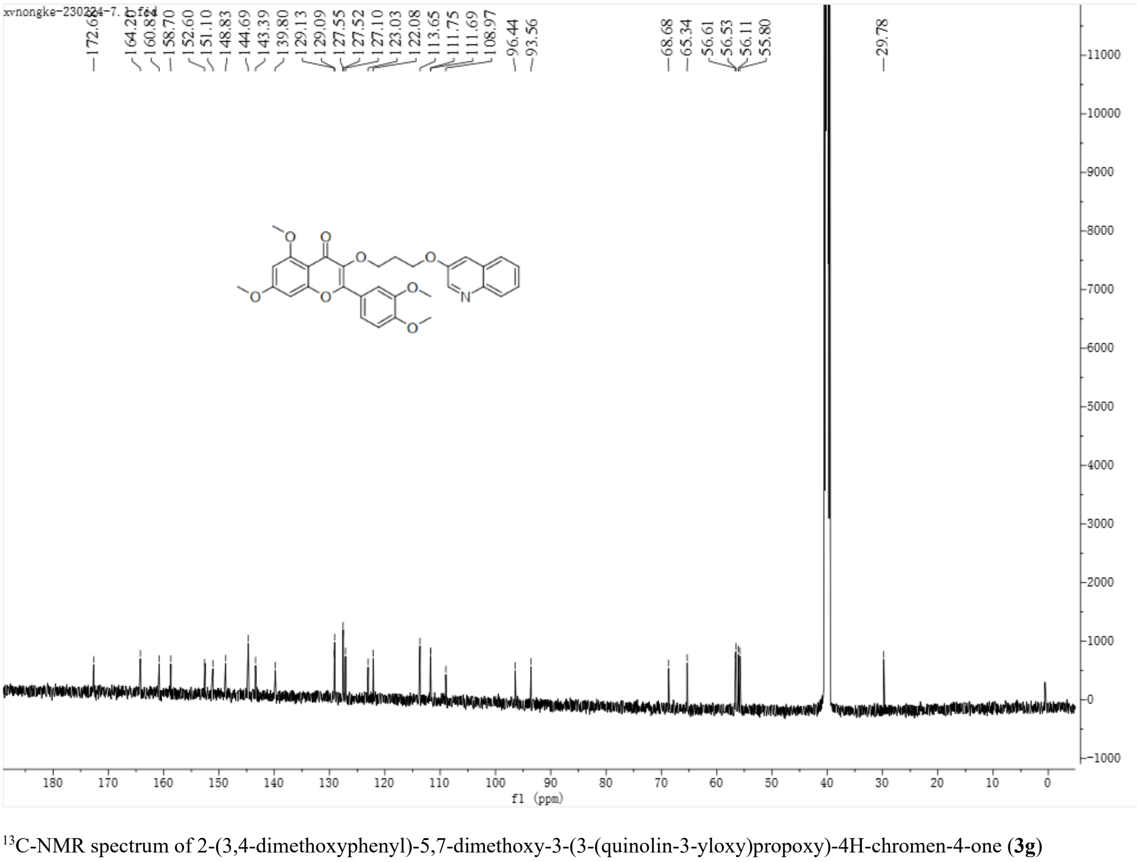

^{13}C-NMR spectrum of 2-(3,4-dimethoxyphenyl)-5,7-dimethoxy-3-(3-(quinolin-3-yloxy)propoxy)-4H-chromen-4-one (**3g**)

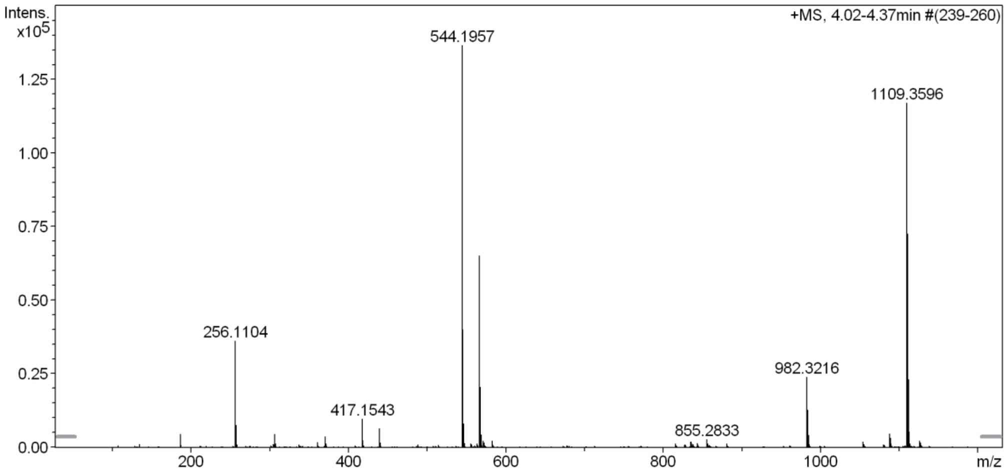

HRMS spectrum of 2-(3,4-dimethoxyphenyl)-5,7-dimethoxy-3-(3-(quinolin-3-yloxy)propoxy)-4H-chromen-4-one (**3g**)

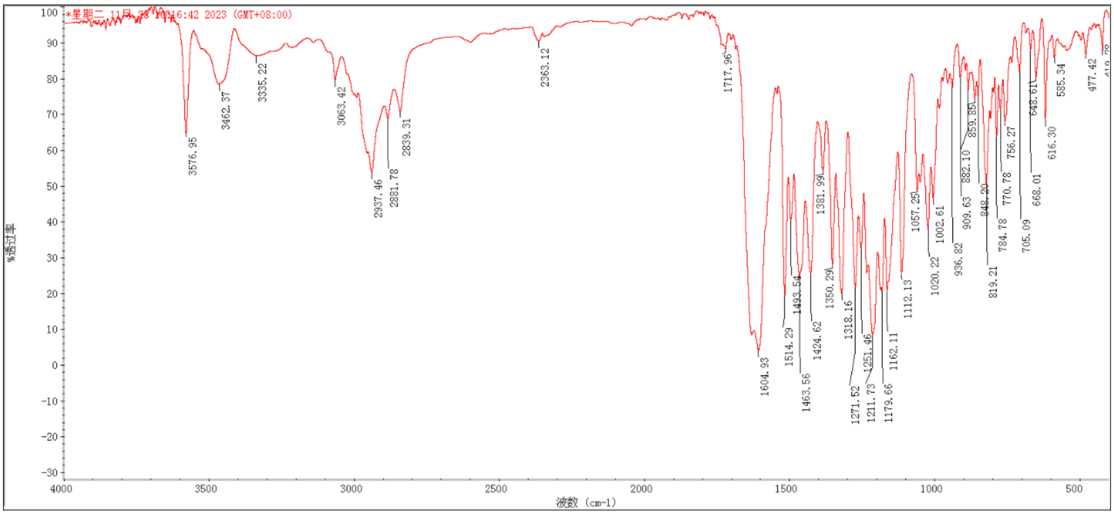

IR spectra of 2-(3,4-dimethoxyphenyl)-5,7-dimethoxy-3-(3-(quinolin-3-yloxy)propoxy)-4H-chromen-4-one (**3g**)

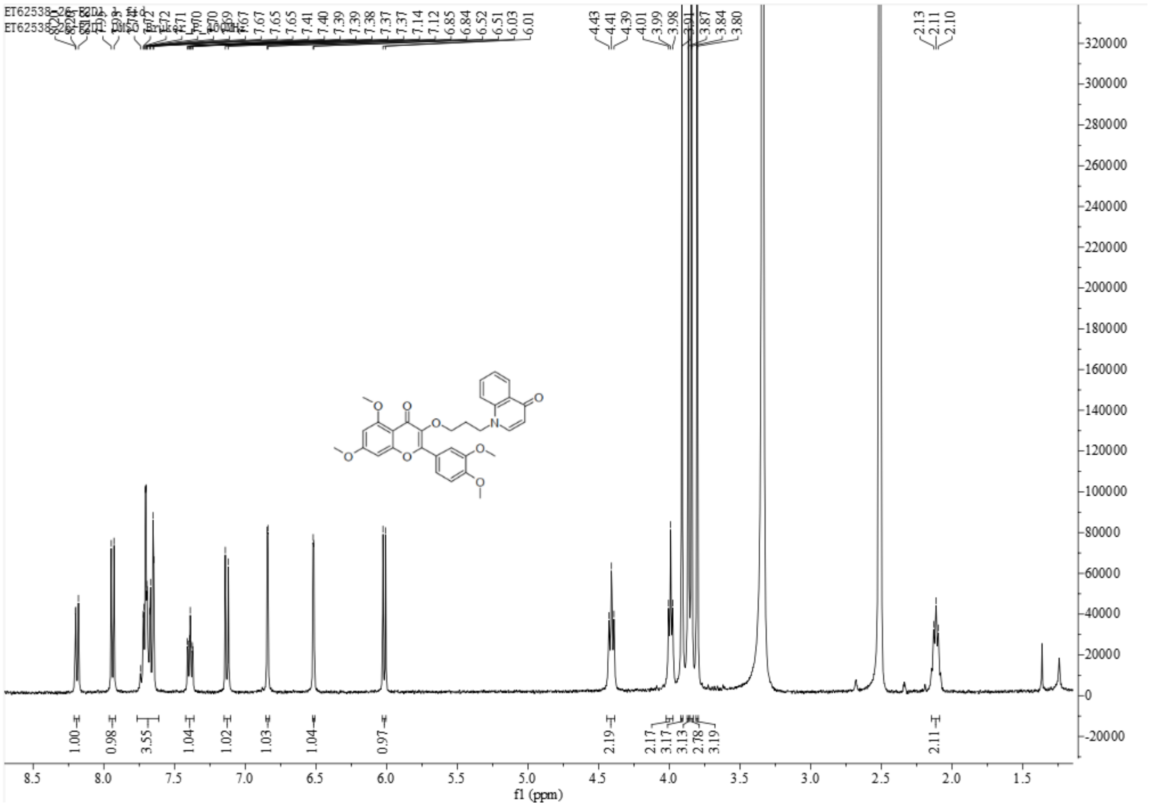

^{1}H-NMR spectrum of 2-(3,4-dimethoxyphenyl)-5,7-dimethoxy-3-(3-(quinolin-4-yloxy)propoxy)-4H-chromen-4-one (**3h**)

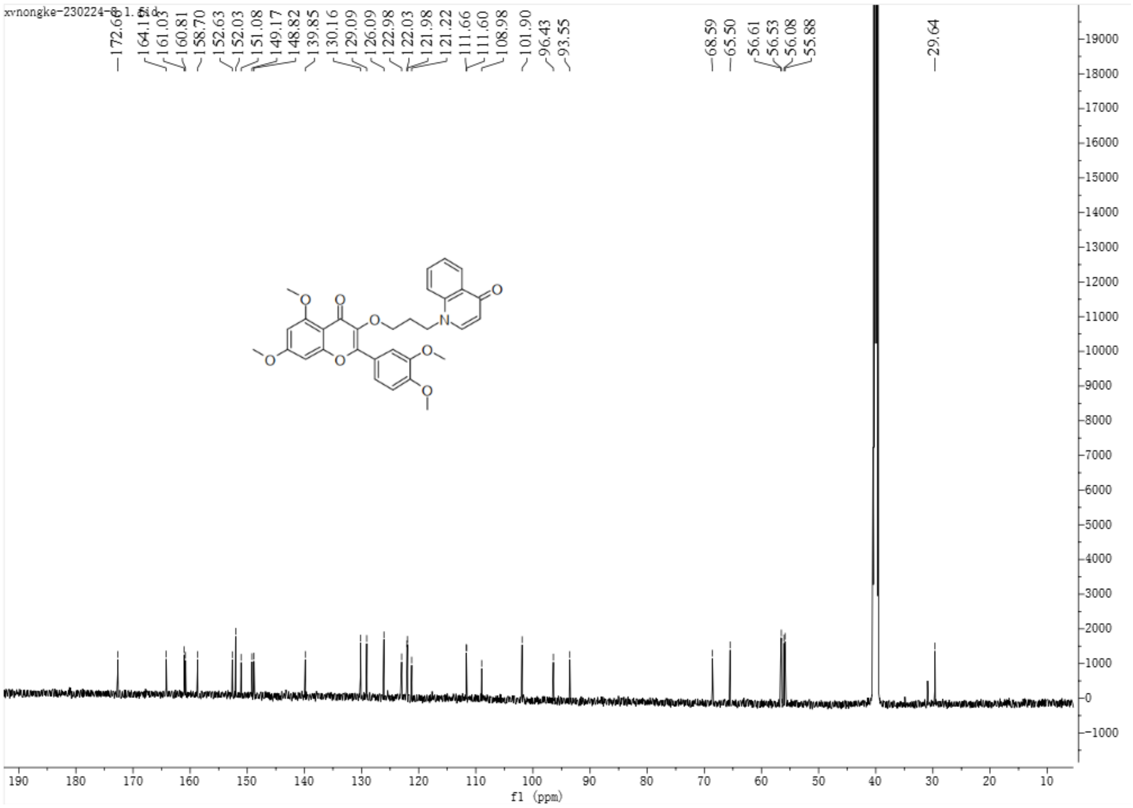

^{13}C-NMR spectrum of 2-(3,4-dimethoxyphenyl)-5,7-dimethoxy-3-(3-(quinolin-4-yloxy)propoxy)-4H-chromen-4-one (**3h**)

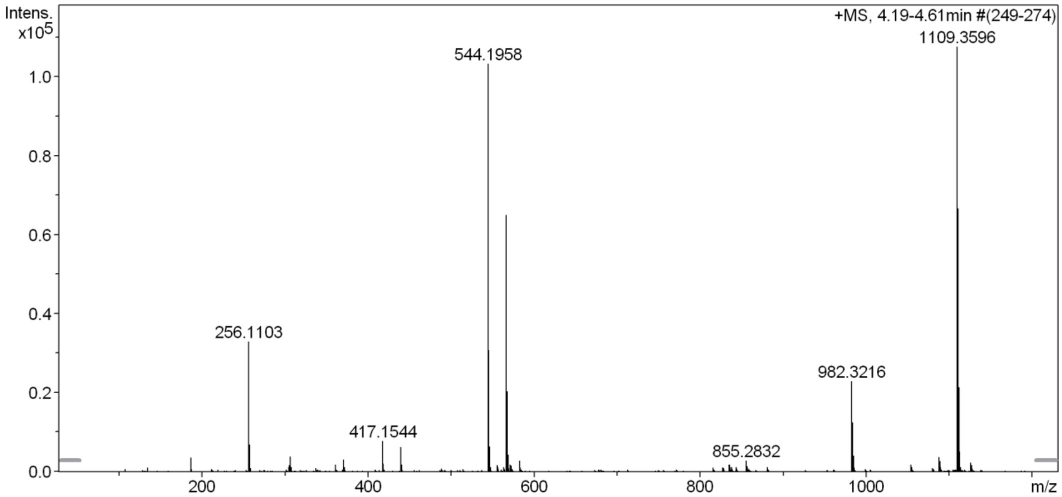

HRMS spectrum of 2-(3,4-dimethoxyphenyl)-5,7-dimethoxy-3-(3-(quinolin-4-yloxy)propoxy)-4H-chromen-4-one (**3h**)

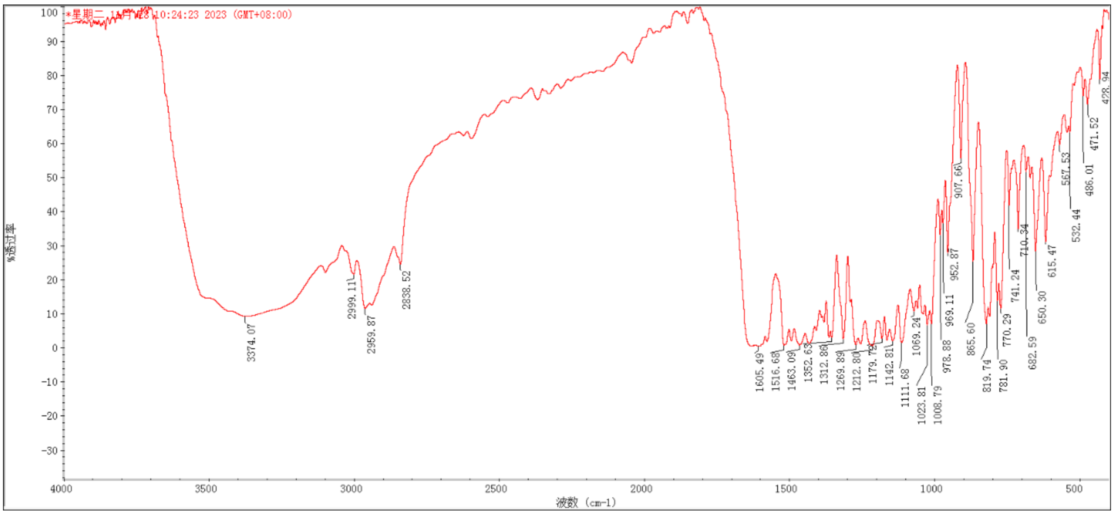

IR spectra of 2-(3,4-dimethoxyphenyl)-5,7-dimethoxy-3-(3-(quinolin-4-yloxy)propoxy)-4H-chromen-4-one (**3h**)

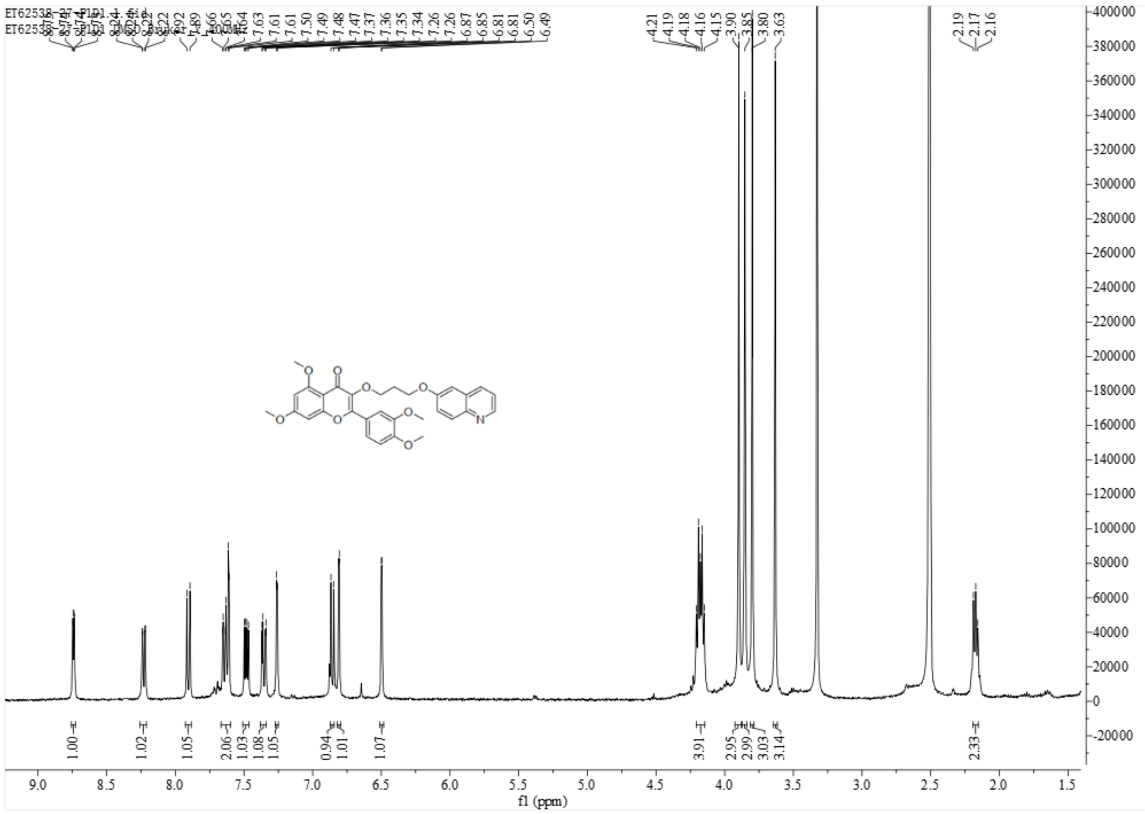

^{1}H-NMR spectrum of 2-(3,4-dimethoxyphenyl)-5,7-dimethoxy-3-(3-(quinolin-6-yloxy)propoxy)-4H-chromen-4-one (**3i**)

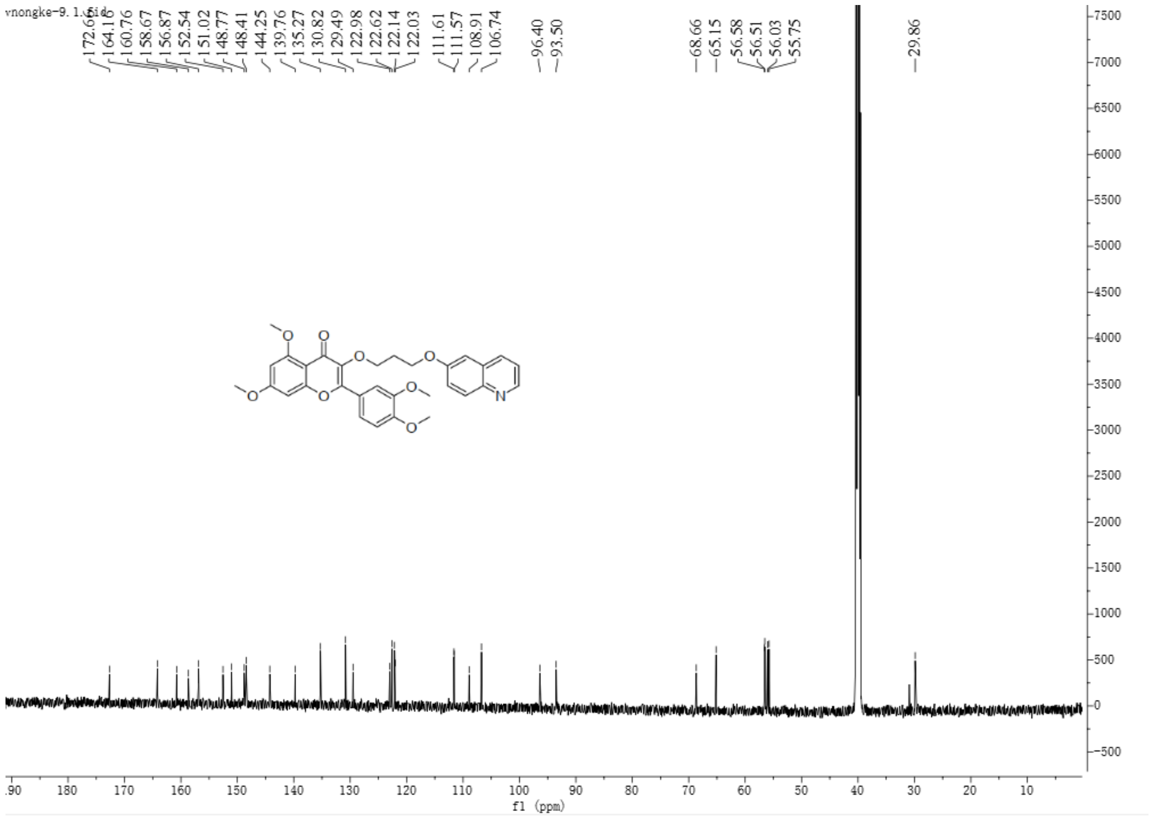

^{13}C-NMR spectrum of 2-(3,4-dimethoxyphenyl)-5,7-dimethoxy-3-(3-(quinolin-6-yloxy)propoxy)-4H-chromen-4-one (**3i**)

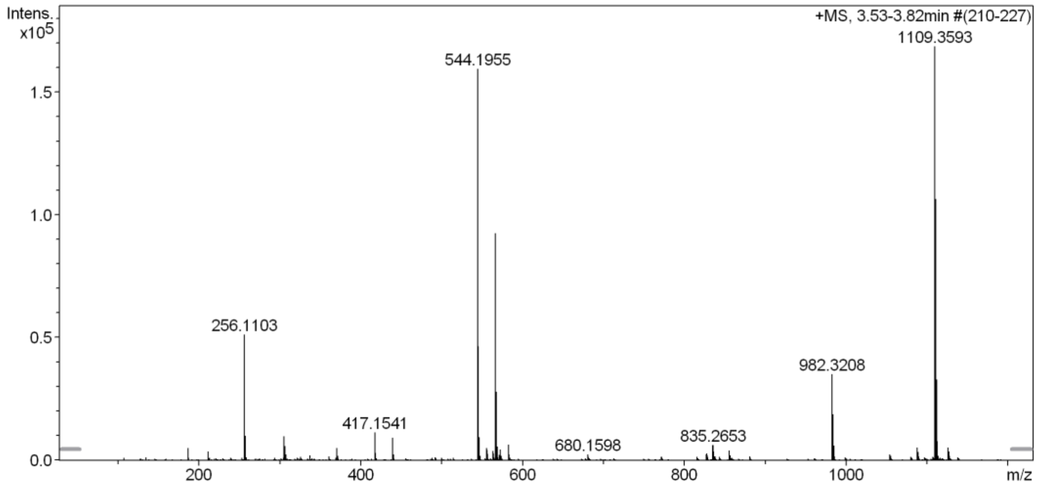

HRMS spectrum of 2-(3,4-dimethoxyphenyl)-5,7-dimethoxy-3-(3-(quinolin-6-yloxy)propoxy)-4H-chromen-4-one (**3i**)

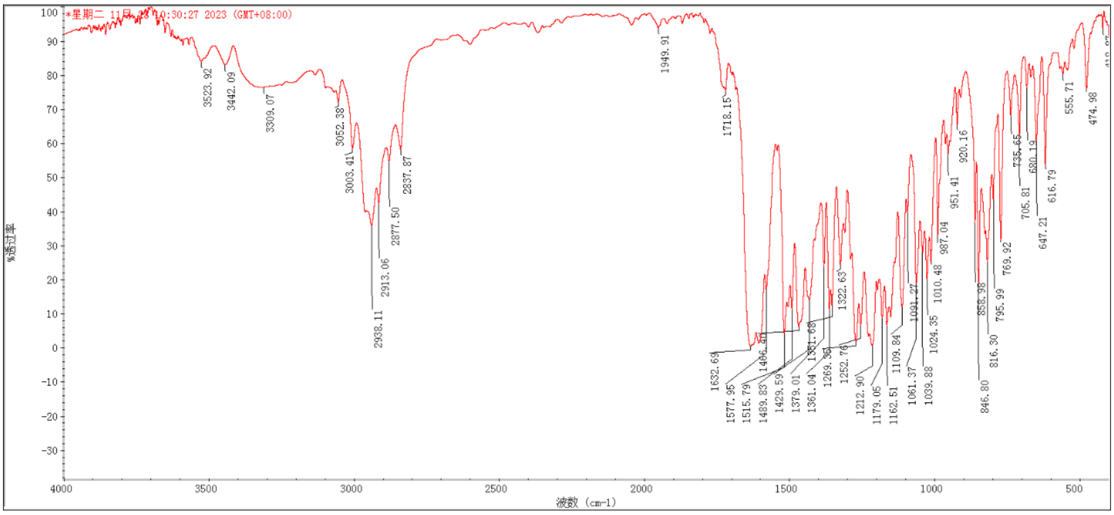

IR spectra of 2-(3,4-dimethoxyphenyl)-5,7-dimethoxy-3-(3-(quinolin-6-yloxy)propoxy)-4H-chromen-4-one (**3i**)

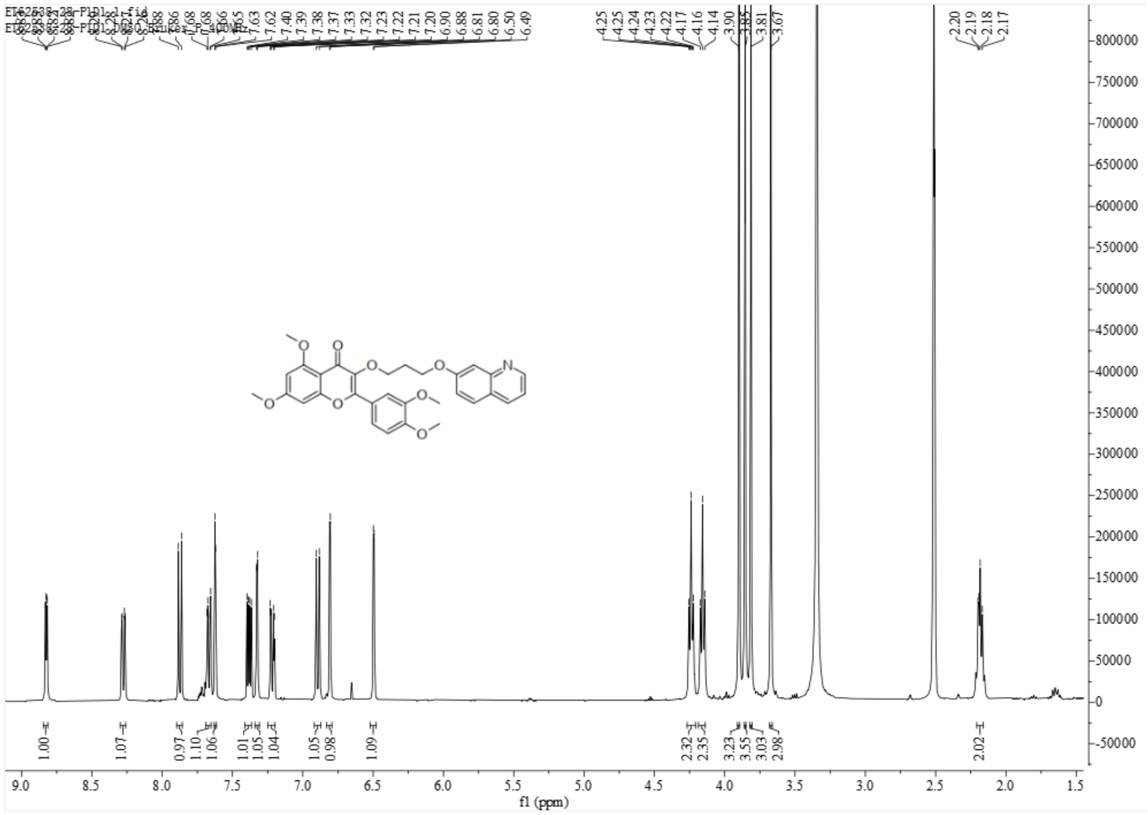

^{1}H-NMR spectrum of 2-(3,4-dimethoxyphenyl)-5,7-dimethoxy-3-(3-(quinolin-7-yloxy)propoxy)-4H-chromen-4-one (**3j**)

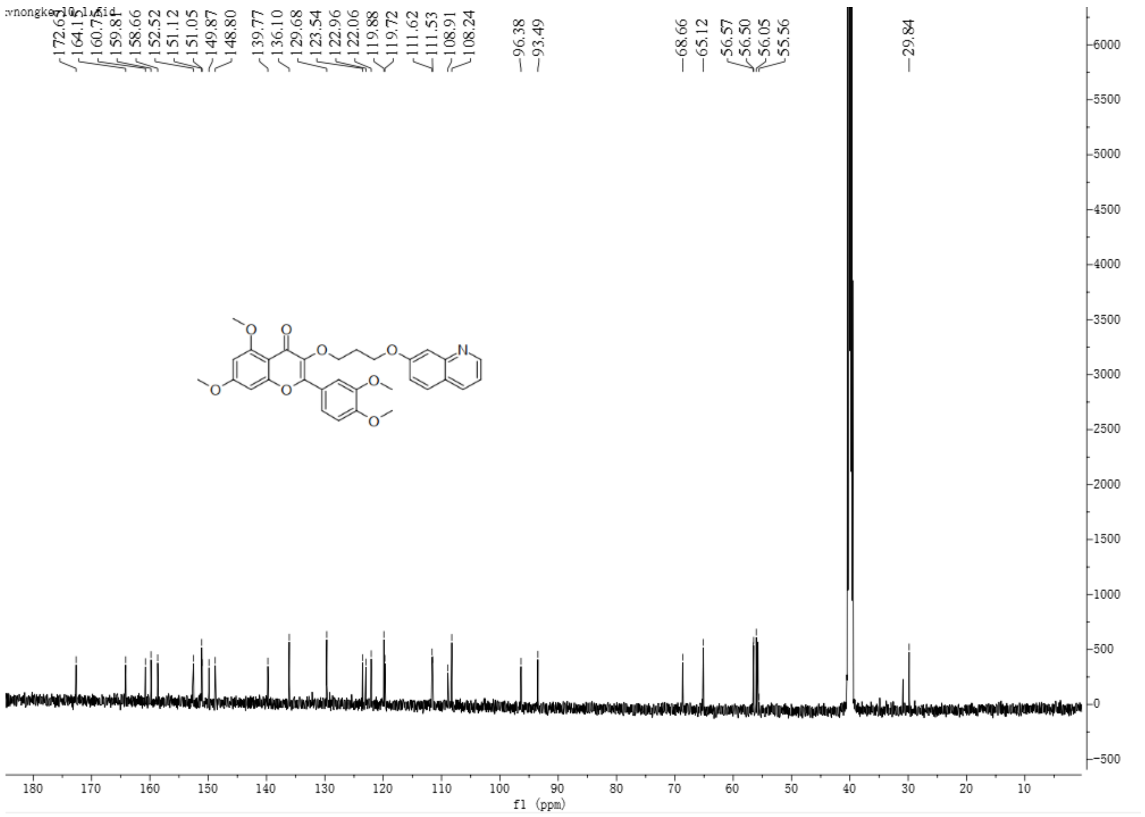

^{13}C-NMR spectrum of 2-(3,4-dimethoxyphenyl)-5,7-dimethoxy-3-(3-(quinolin-7-yloxy)propoxy)-4H-chromen-4-one (**3j**)

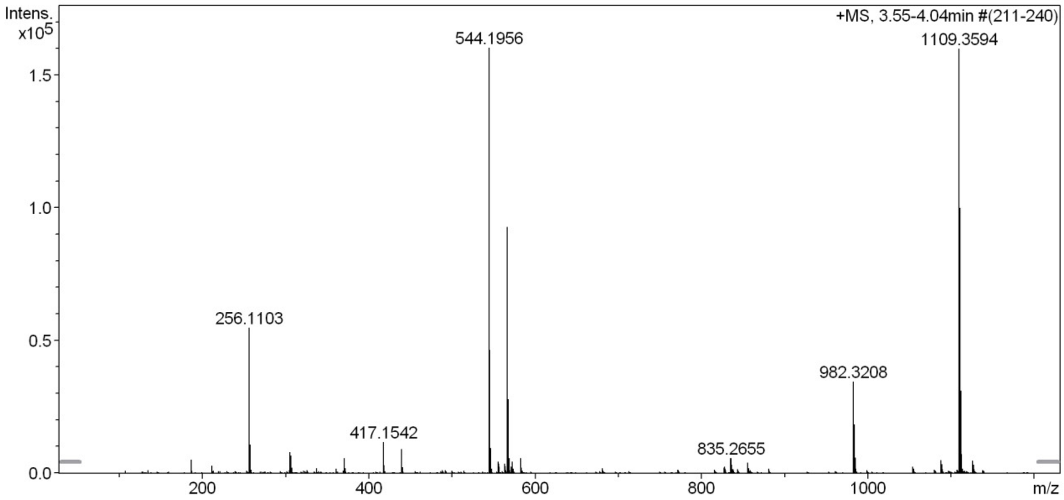

HRMS spectrum of 2-(3,4-dimethoxyphenyl)-5,7-dimethoxy-3-(3-(quinolin-7-yloxy)propoxy)-4H-chromen-4-one (**3j**)

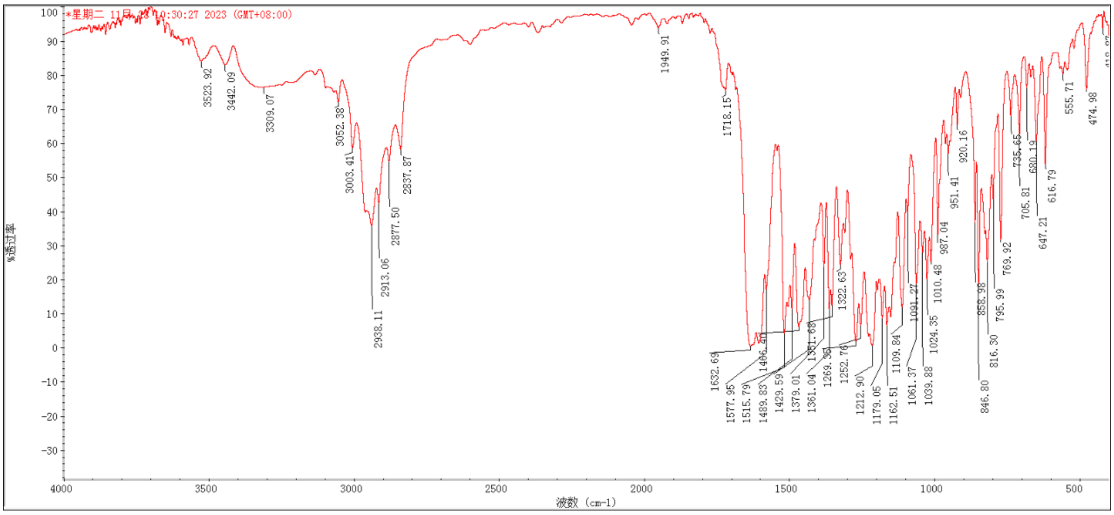

IR spectra of 2-(3,4-dimethoxyphenyl)-5,7-dimethoxy-3-(3-(quinolin-7-yloxy)propoxy)-4H-chromen-4-one (**3j**)

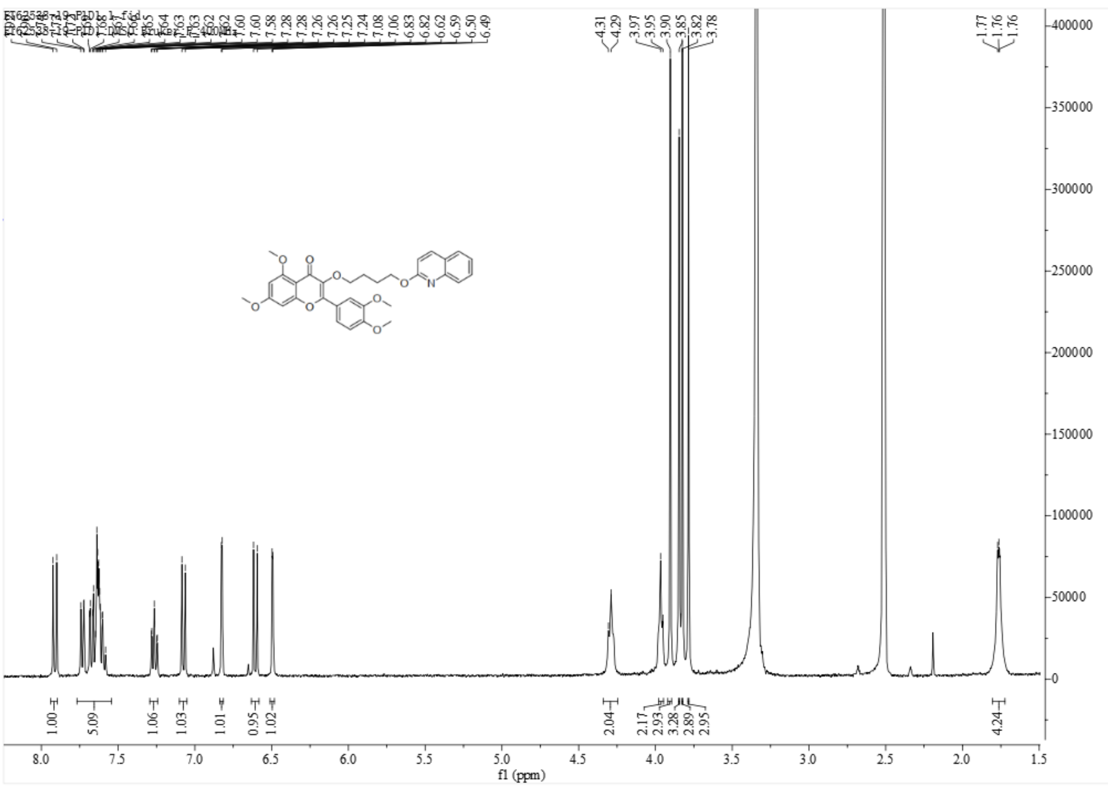

^{1}H-NMR spectrum of 2-(3,4-dimethoxyphenyl)-5,7-dimethoxy-3-(4-(quinolin-2-yloxy)butoxy)-4H-chromen-4-one (**3k**)

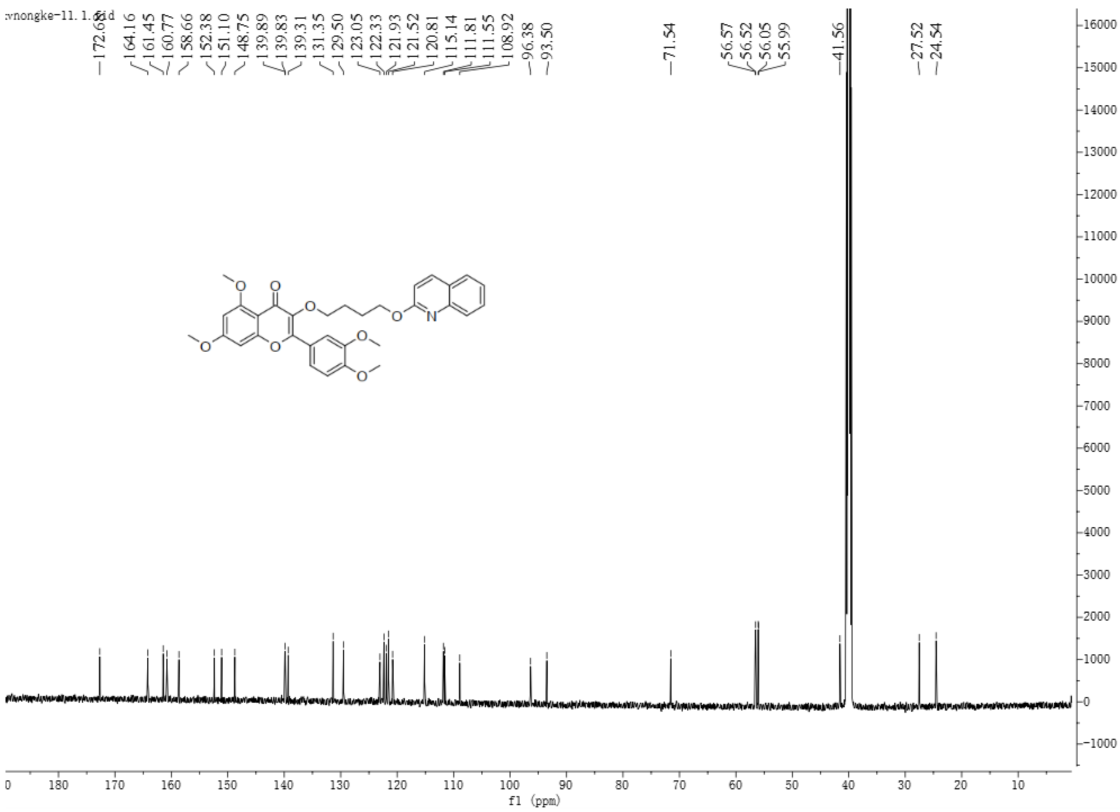

^{13}C-NMR spectrum of 2-(3,4-dimethoxyphenyl)-5,7-dimethoxy-3-(4-(quinolin-2-yloxy)butoxy)-4H-chromen-4-one (**3k**)

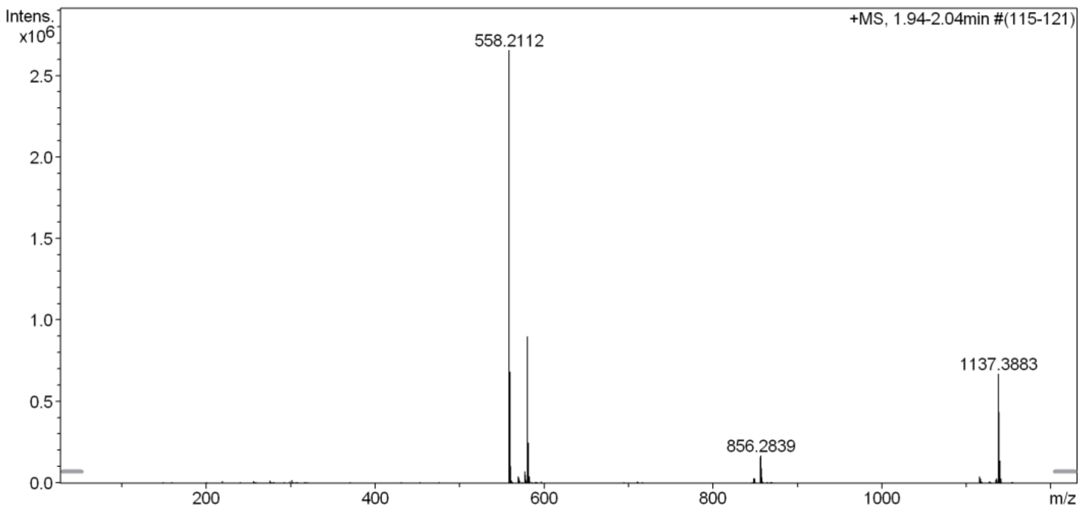

HRMS spectrum of 2-(3,4-dimethoxyphenyl)-5,7-dimethoxy-3-(4-(quinolin-2-yloxy)butoxy)-4H-chromen-4-one (**3k**)

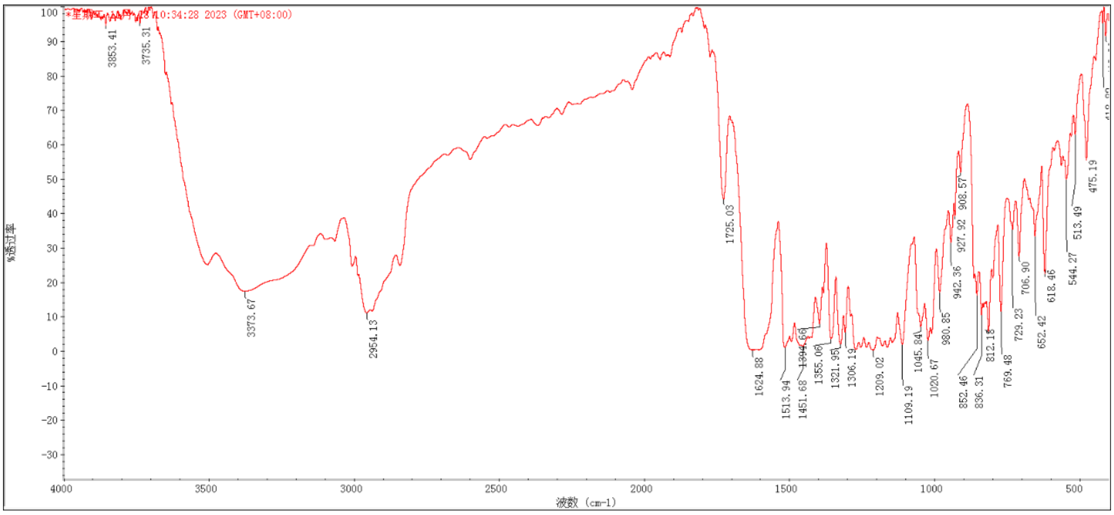

IR spectra of 2-(3,4-dimethoxyphenyl)-5,7-dimethoxy-3-(4-(quinolin-2-yloxy)butoxy)-4H-chromen-4-one (**3k**)

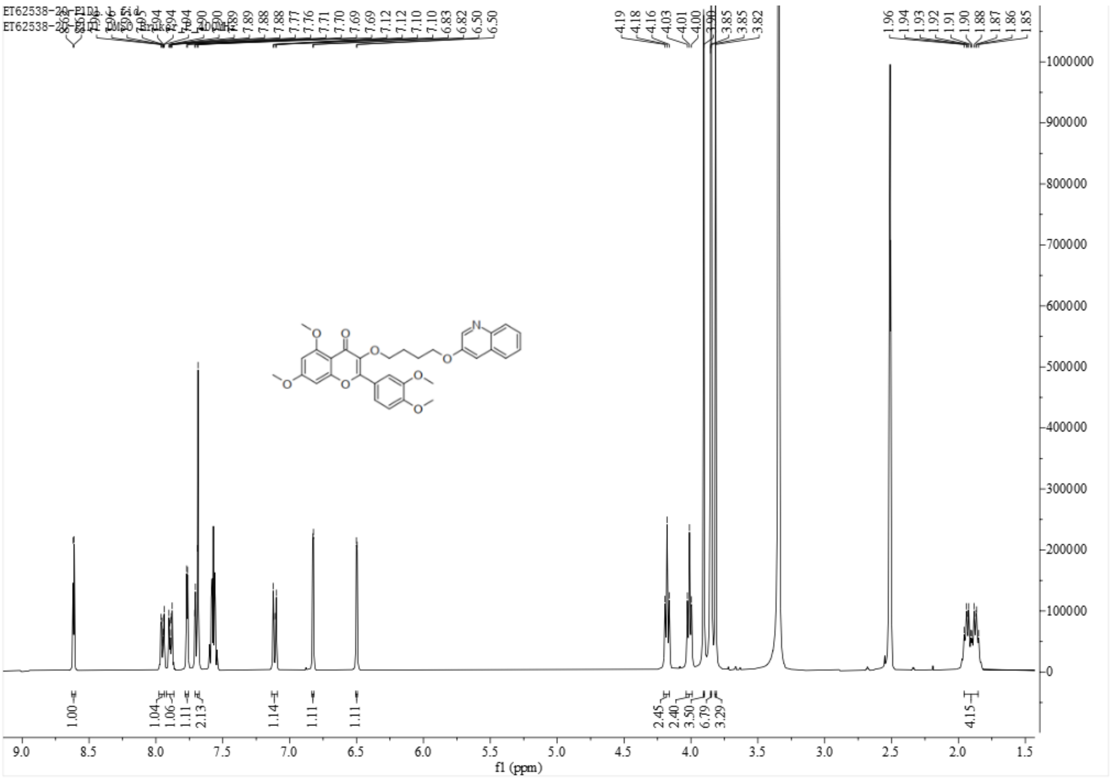

^{13}C-NMR spectrum of 2-(3,4-dimethoxyphenyl)-5,7-dimethoxy-3-(4-(quinolin-3-yloxy)butoxy)-4H-chromen-4-one (**3l**)

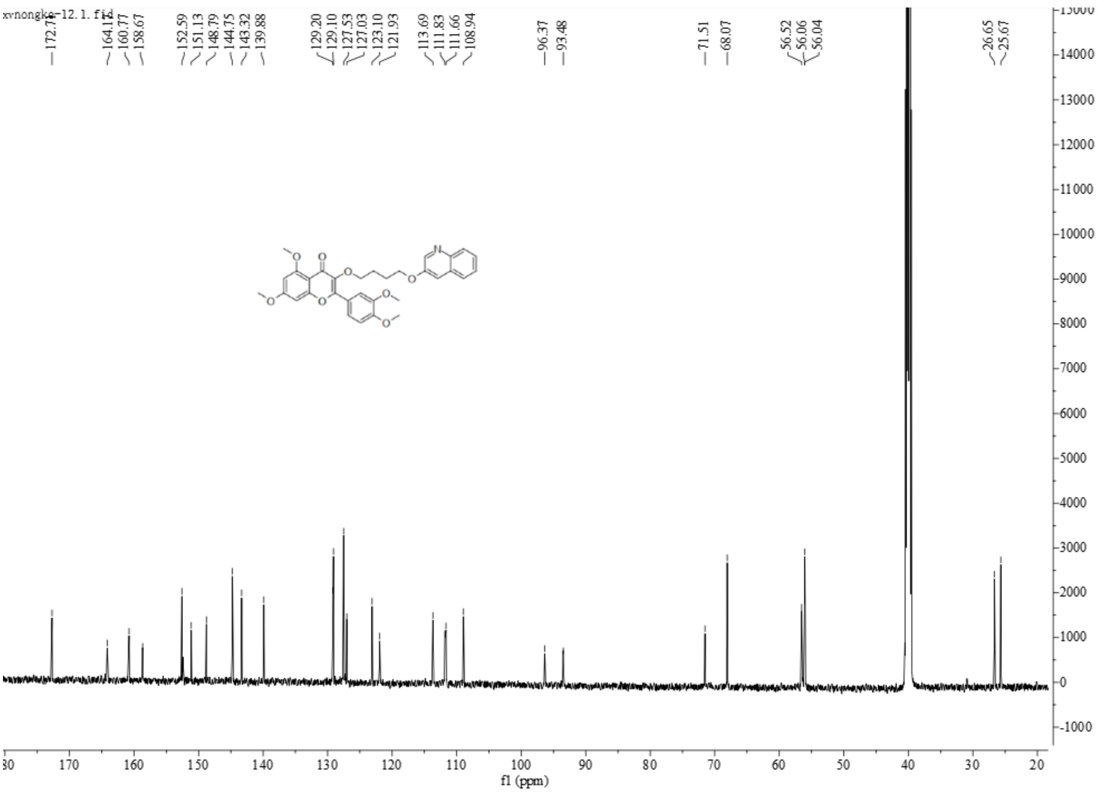

^{13}C-NMR spectrum of 2-(3,4-dimethoxyphenyl)-5,7-dimethoxy-3-(4-(quinolin-3-yloxy)butoxy)-4H-chromen-4-one (**3l**)

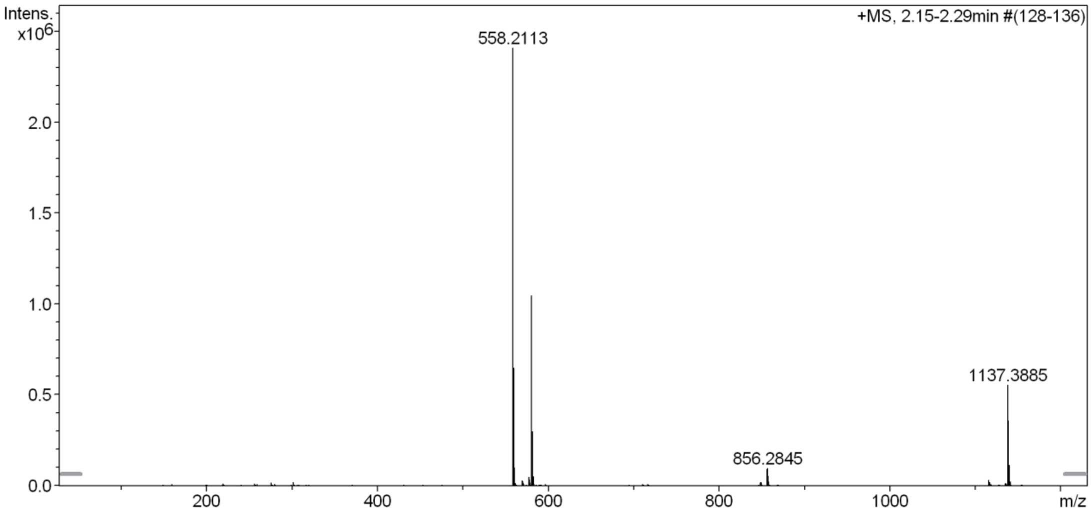

HRMS spectrum of 2-(3,4-dimethoxyphenyl)-5,7-dimethoxy-3-(4-(quinolin-3-yloxy)butoxy)-4H-chromen-4-one (**3l**)

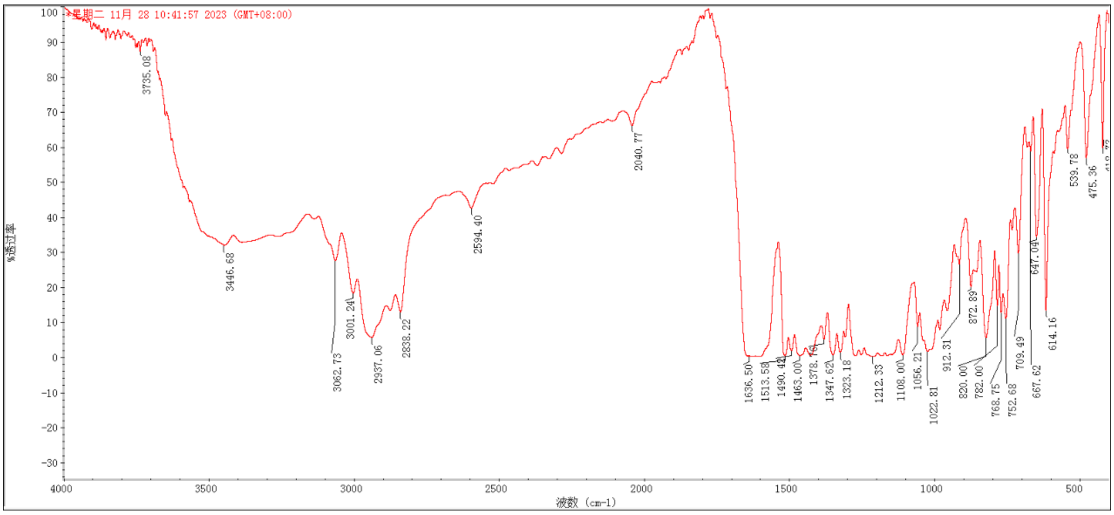

IR spectra of 2-(3,4-dimethoxyphenyl)-5,7-dimethoxy-3-(4-(quinolin-3-yloxy)butoxy)-4H-chromen-4-one (**3l**)

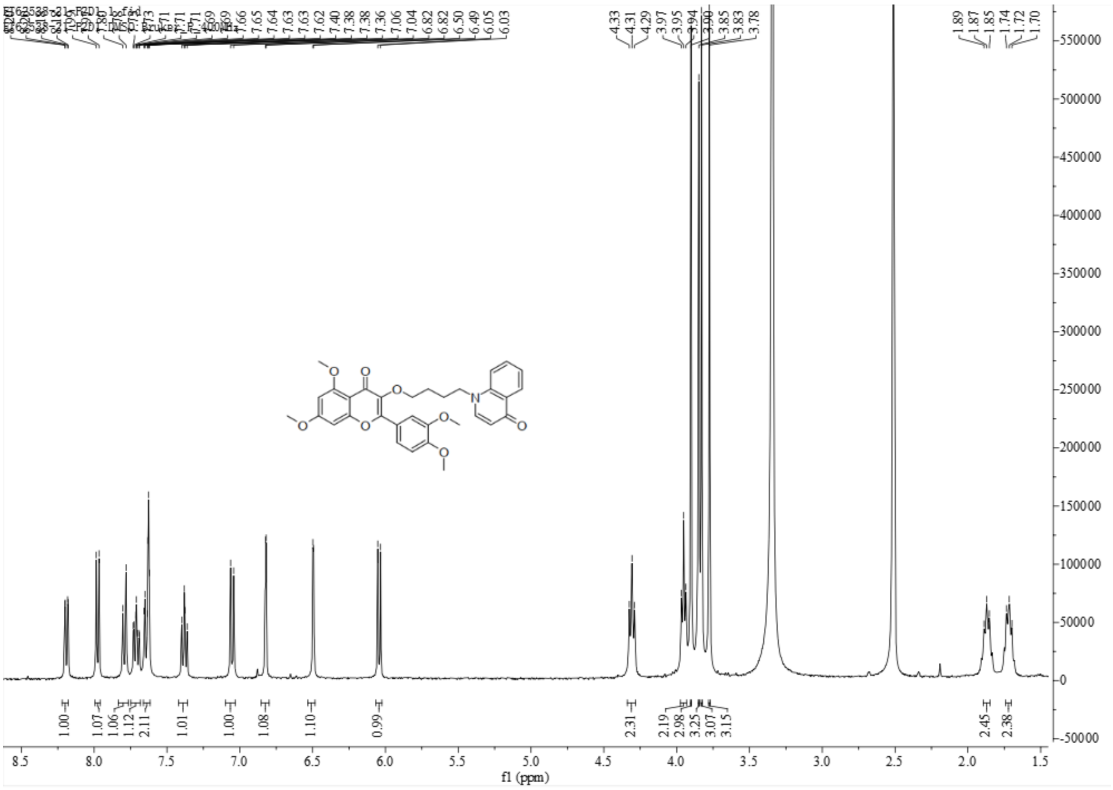

^{1}H-NMR spectrum of 2-(3,4-dimethoxyphenyl)-5,7-dimethoxy-3-(4-(quinolin-4-yloxy)butoxy)-4H-chromen-4-one (**3m**)

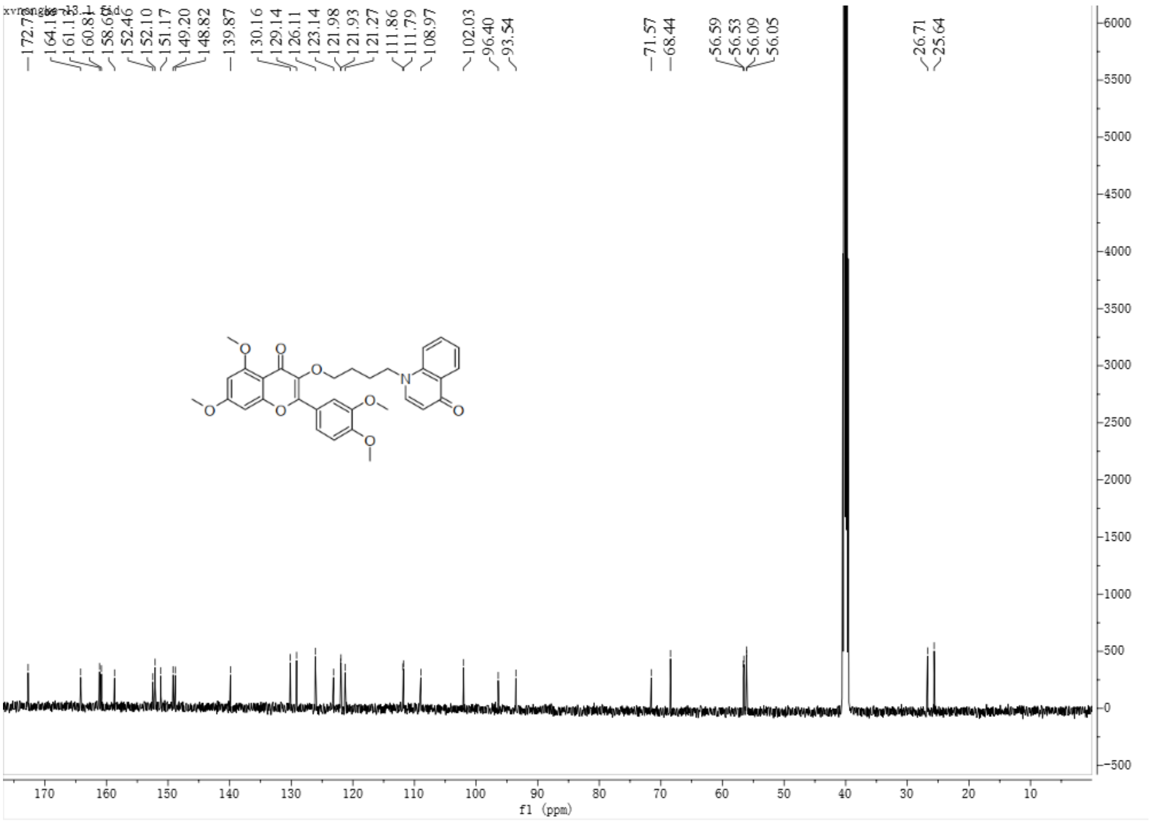

^{13}C-NMR spectrum of 2-(3,4-dimethoxyphenyl)-5,7-dimethoxy-3-(4-(quinolin-4-yloxy)butoxy)-4H-chromen-4-one (**3m**)

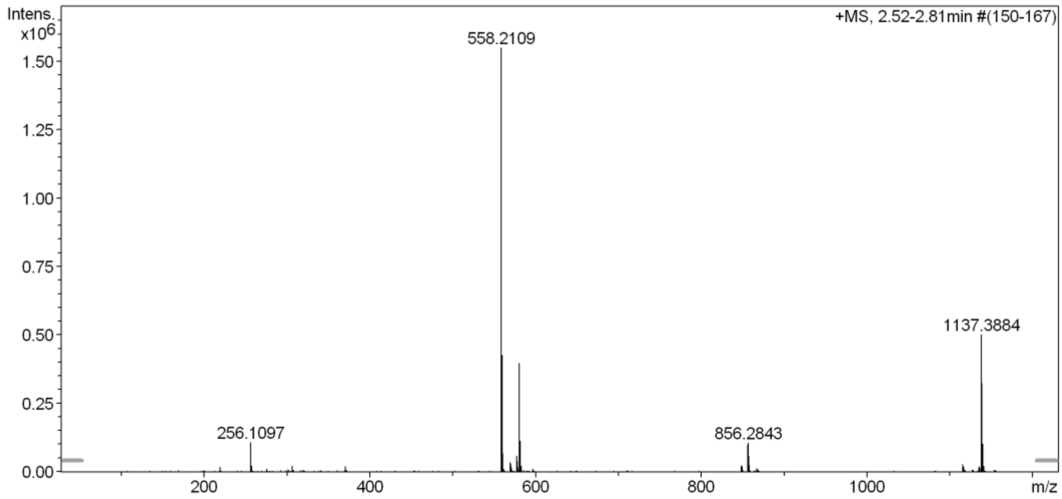

HRMS spectrum of 2-(3,4-dimethoxyphenyl)-5,7-dimethoxy-3-(4-(quinolin-4-yloxy)butoxy)-4H-chromen-4-one (**3m**)

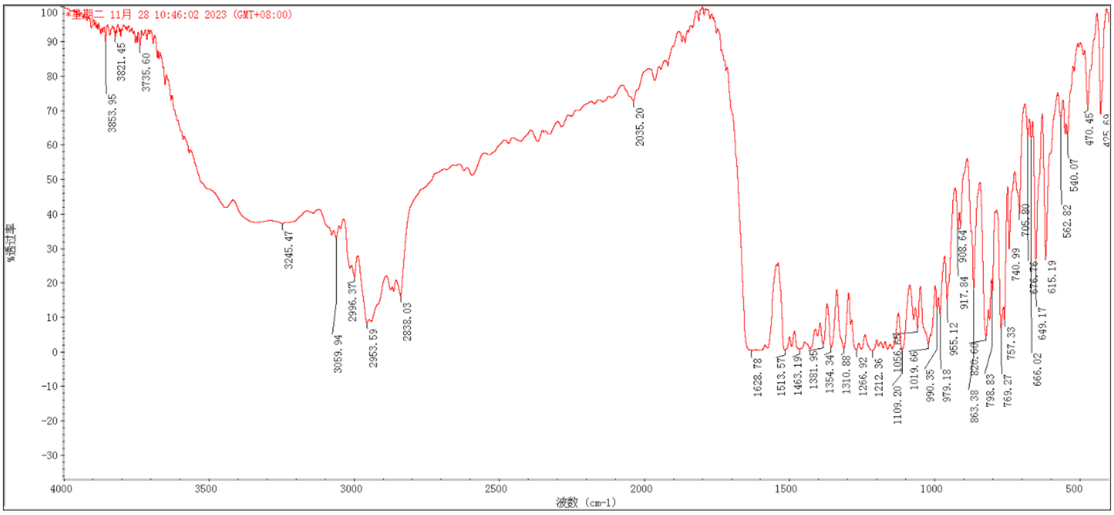

IR spectra of 2-(3,4-dimethoxyphenyl)-5,7-dimethoxy-3-(4-(quinolin-4-yloxy)butoxy)-4H-chromen-4-one (**3m**)

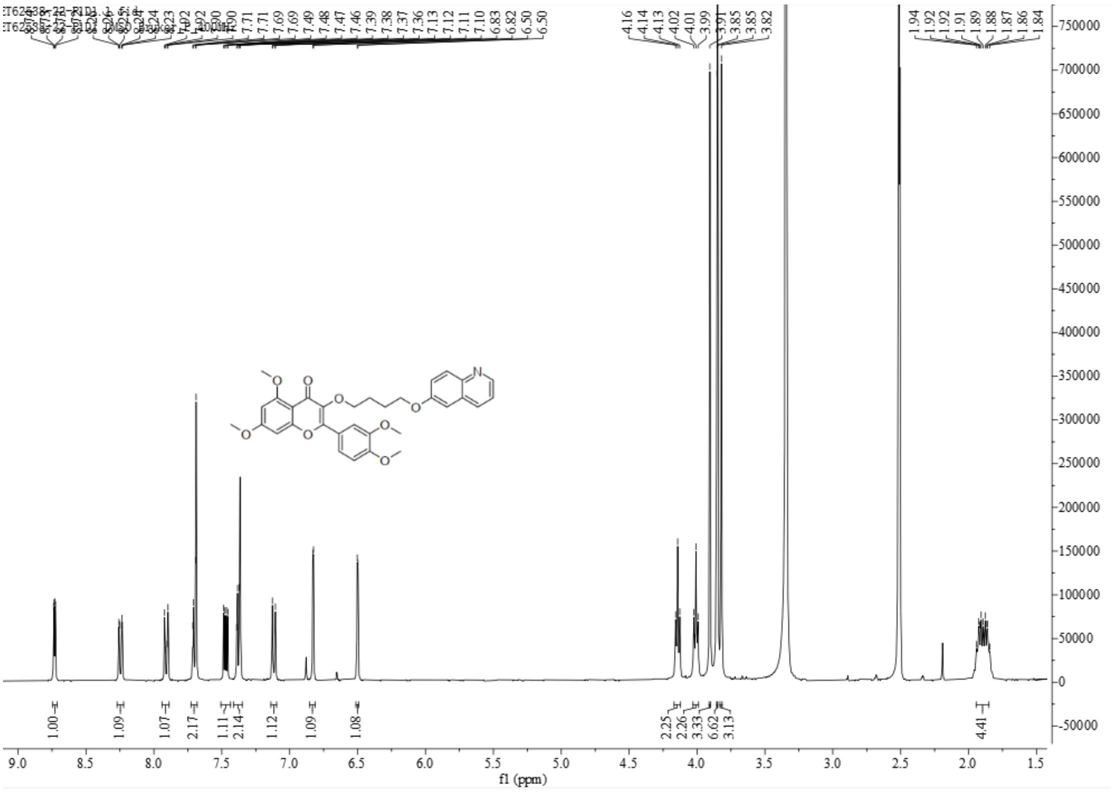

^{1}H-NMR spectrum of 2-(3,4-dimethoxyphenyl)-5,7-dimethoxy-3-(4-(quinolin-6-yloxy)butoxy)-4H-chromen-4-one (**3n**)

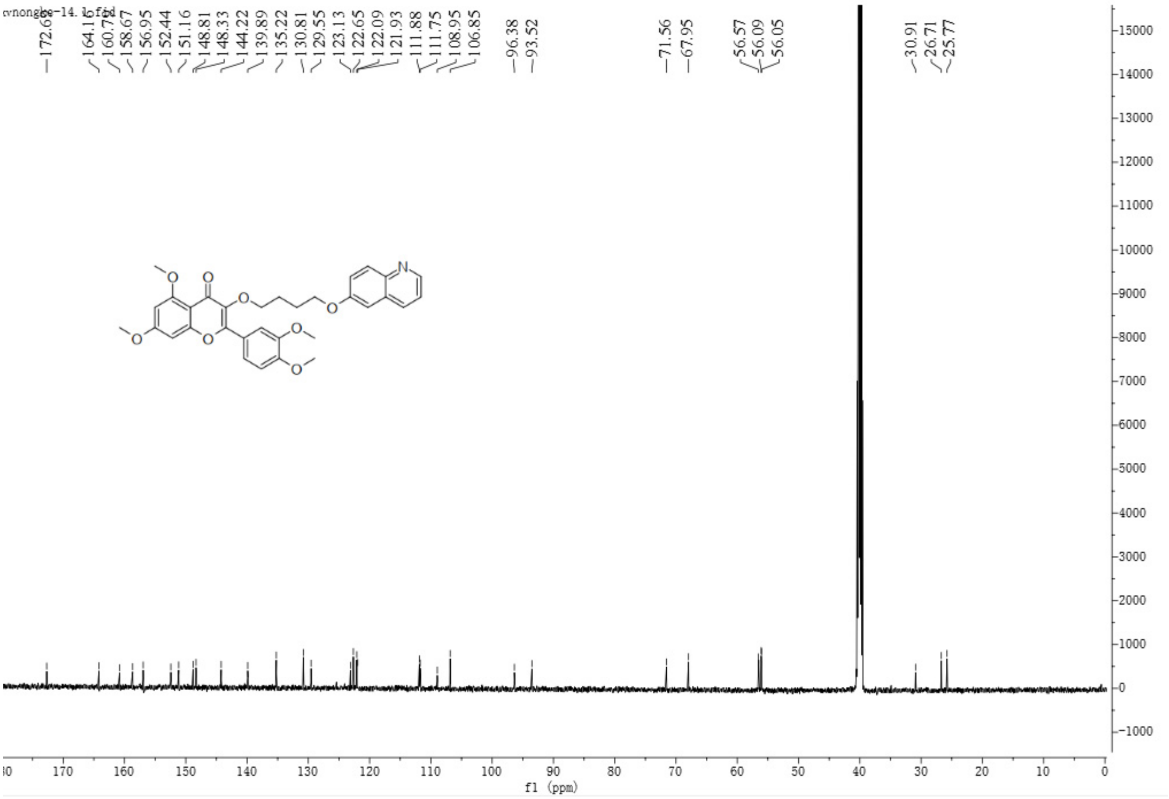

^{13}C-NMR spectrum of 2-(3,4-dimethoxyphenyl)-5,7-dimethoxy-3-(4-(quinolin-6-yloxy)butoxy)-4H-chromen-4-one (**3n**)

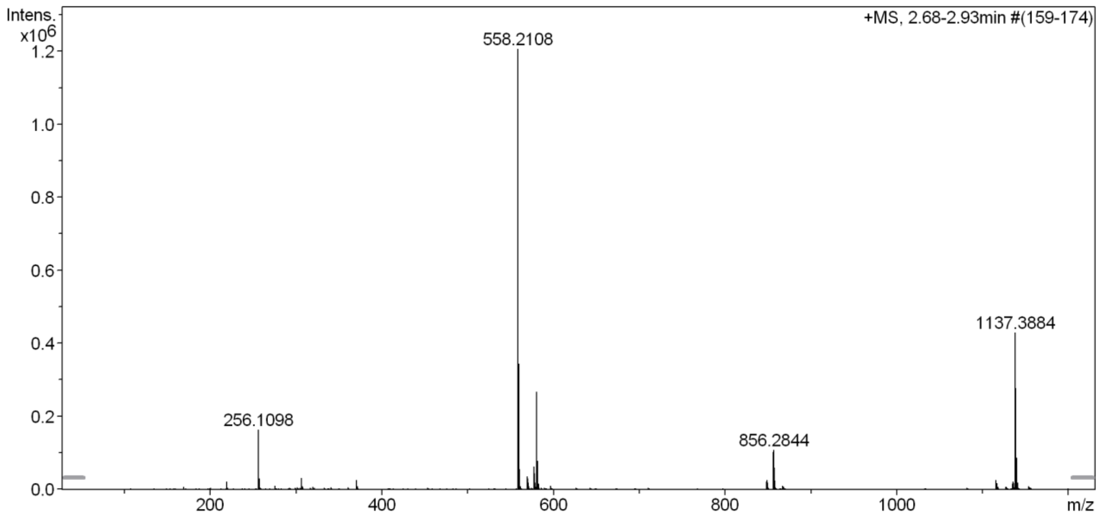

HRMS spectrum of 2-(3,4-dimethoxyphenyl)-5,7-dimethoxy-3-(4-(quinolin-6-yloxy)butoxy)-4H-chromen-4-one (**3n**)

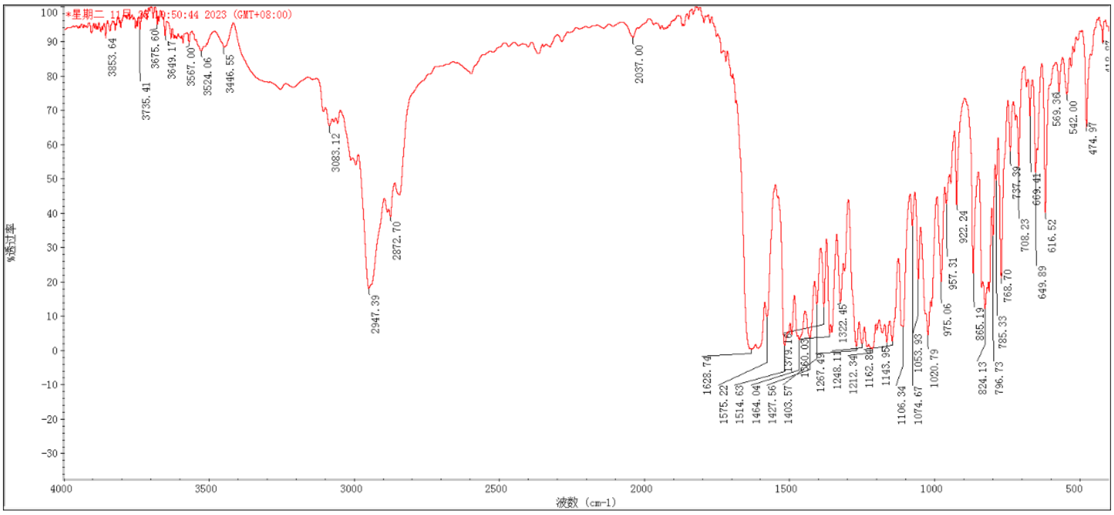

IR spectra of 2-(3,4-dimethoxyphenyl)-5,7-dimethoxy-3-(4-(quinolin-6-yloxy)butoxy)-4H-chromen-4-one (**3n**)

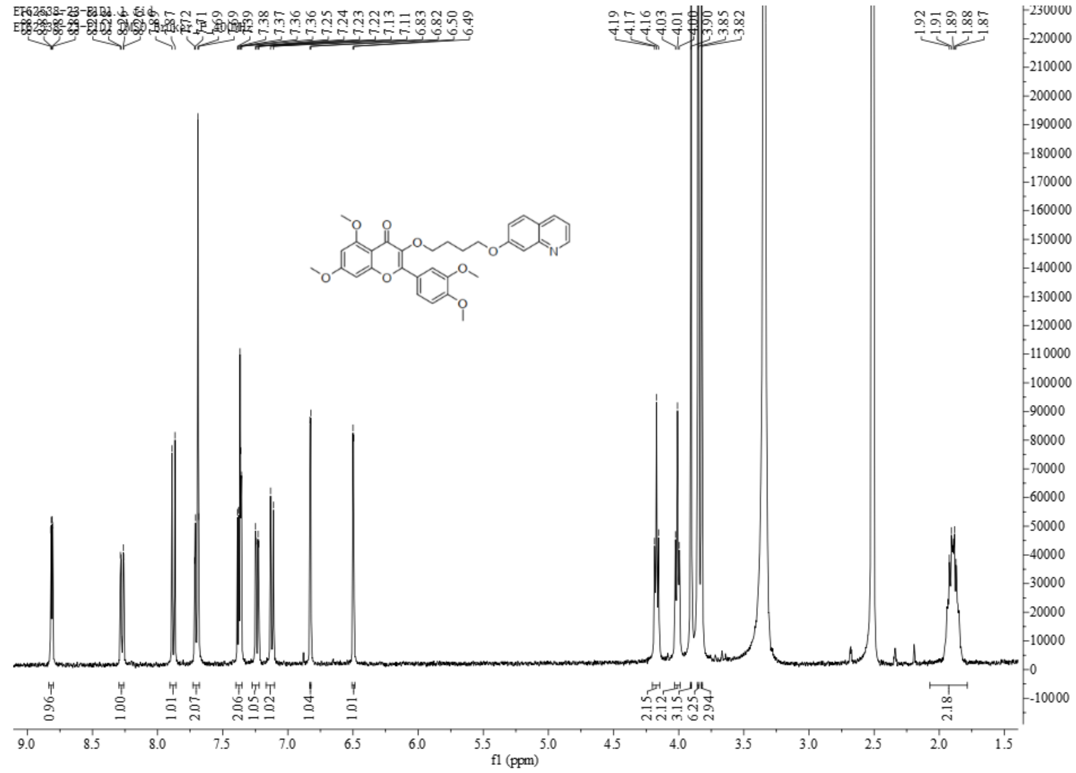

^{1}H-NMR spectrum of 2-(3,4-dimethoxyphenyl)-5,7-dimethoxy-3-(4-(quinolin-7-yloxy)butoxy)-4H-chromen-4-one (**3o**)

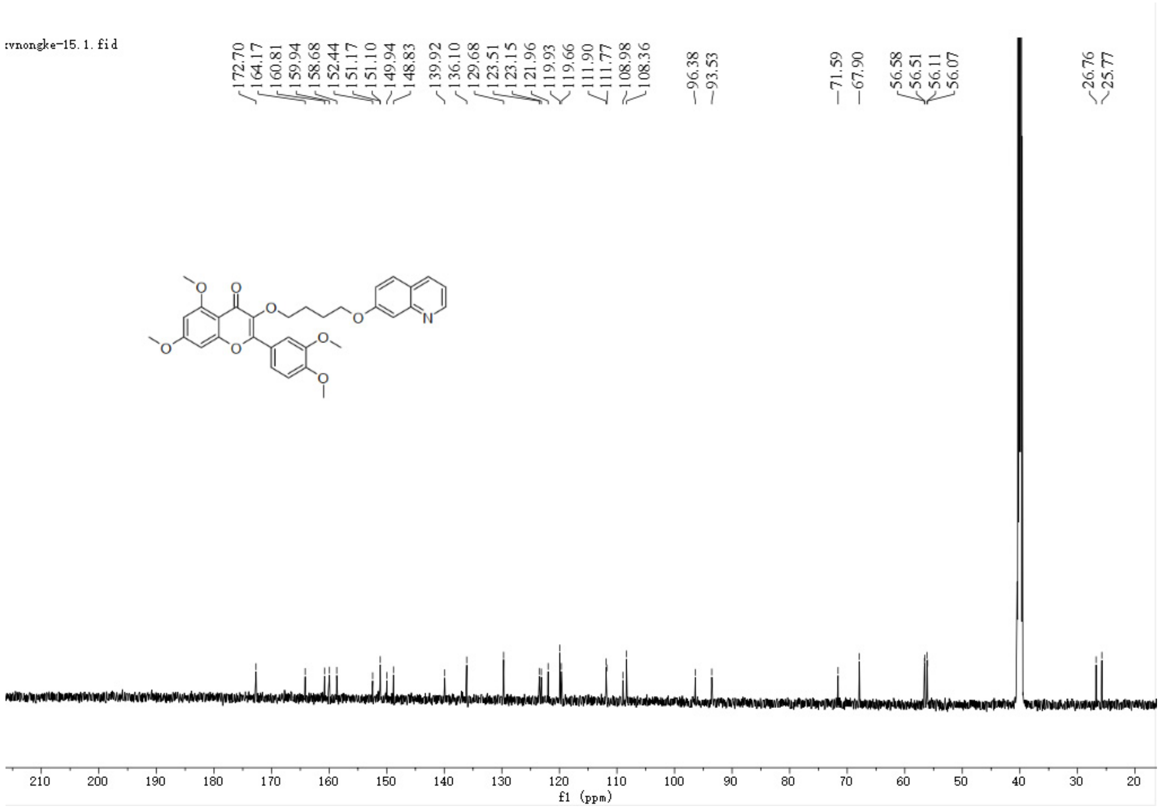

^{13}C-NMR spectrum of 2-(3,4-dimethoxyphenyl)-5,7-dimethoxy-3-(4-(quinolin-7-yloxy)butoxy)-4H-chromen-4-one (**3o**)

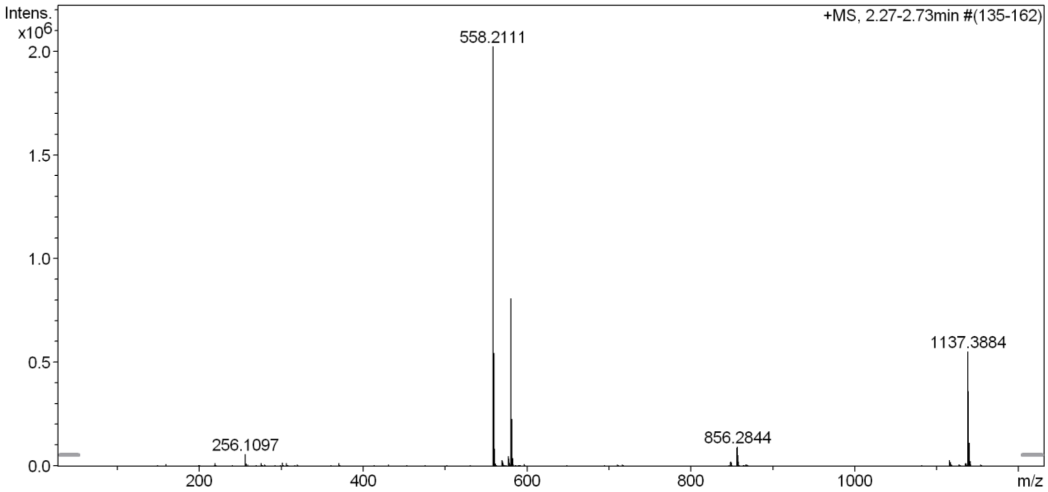

HRMS spectrum of 2-(3,4-dimethoxyphenyl)-5,7-dimethoxy-3-(4-(quinolin-7-yloxy)butoxy)-4H-chromen-4-one (**3o**)

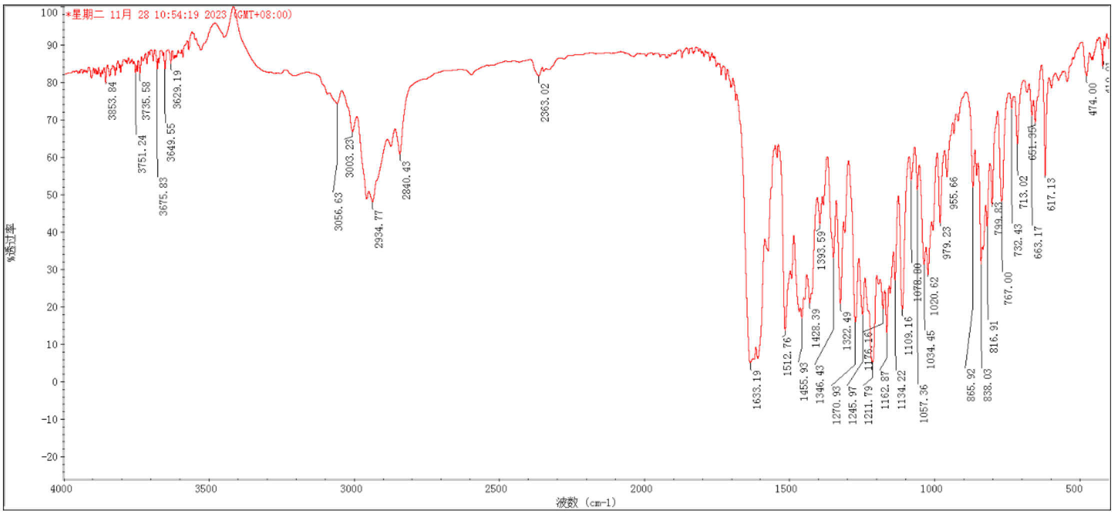

IR spectra of 2-(3,4-dimethoxyphenyl)-5,7-dimethoxy-3-(4-(quinolin-7-yloxy)butoxy)-4H-chromen-4-one (**3o**)
